# Machine-learning analysis reveals an important role for negative selection in shaping cancer aneuploidy landscapes

**DOI:** 10.1186/s13059-024-03225-7

**Published:** 2024-04-15

**Authors:** Juman Jubran, Rachel Slutsky, Nir Rozenblum, Lior Rokach, Uri Ben-David, Esti Yeger-Lotem

**Affiliations:** 1 Department of Clinical Biochemistry and Pharmacology, Ben-Gurion University of the Negev, 84105 Beer Sheva, Israel; 2 Department of Human Molecular Genetics and Biochemistry, Faculty of Medicine, Tel Aviv University, Tel Aviv, Israel; 3 Department of Software & Information Systems Engineering, Ben-Gurion University of the Negev, 84105 Beer Sheva, Israel; 4 The National Institute for Biotechnology in the Negev, Ben-Gurion University of the Negev, 84105 Beer Sheva, Israel

## Abstract

**Background:**

Aneuploidy, an abnormal number of chromosomes within a cell, is a hallmark of cancer. Patterns of aneuploidy differ across cancers, yet are similar in cancers affecting closely related tissues. The selection pressures underlying aneuploidy patterns are not fully understood, hindering our understanding of cancer development and progression.

**Results:**

Here, we apply interpretable machine learning methods to study tissue-selective aneuploidy patterns. We define 20 types of features corresponding to genomic attributes of chromosome-arms, normal tissues, primary tumors, and cancer cell lines (CCLs), and use them to model gains and losses of chromosome arms in 24 cancer types. To reveal the factors that shape the tissue-specific cancer aneuploidy landscapes, we interpret the machine learning models by estimating the relative contribution of each feature to the models. While confirming known drivers of positive selection, our quantitative analysis highlights the importance of negative selection for shaping aneuploidy landscapes. This is exemplified by tumor suppressor gene density being a better predictor of gain patterns than oncogene density, and vice versa for loss patterns. We also identify the importance of tissue-selective features and demonstrate them experimentally, revealing *KLF5* as an important driver for chr13q gain in colon cancer. Further supporting an important role for negative selection in shaping the aneuploidy landscapes, we find compensation by paralogs to be among the top predictors of chromosome arm loss prevalence and demonstrate this relationship for one paralog interaction. Similar factors shape aneuploidy patterns in human CCLs, demonstrating their relevance for aneuploidy research.

**Conclusions:**

Our quantitative, interpretable machine learning models improve the understanding of the genomic properties that shape cancer aneuploidy landscapes.

**Supplementary Information:**

The online version contains supplementary material available at 10.1186/s13059-024-03225-7.

## Introduction

Aneuploidy, defined as an abnormal number of chromosomes or chromosome-arms within a cell, is a characteristic trait of human cancer [1]. Aneuploidy is associated with patient prognosis and with response to anticancer therapies [2, 3], indicating that it can play a driving role in tumorigenesis. It is well established that the fitness advantage conferred by specific aneuploidies depends on the genomic, environmental, and developmental contexts [1]. One important cellular context is the cancer tissue of origin; aneuploidy patterns are cancer type-specific, and cancers that originate from related tissues tend to exhibit similar aneuploidy patterns [2, 4, 5]. Nonetheless, the selection pressures that shape the aneuploidy landscapes of human tumors are not fully understood, and it is not clear why some chromosome-arm gains and losses would recur in some tumor types but not in others.

Several non-mutually exclusive explanations have been previously provided in an attempt to explain the tissue selectivity of aneuploidy patterns. First, the densities of oncogenes (OGs) and tumor suppressor genes (TSGs) are enriched in chromosome-arms that tend to be gained or lost, respectively, potentially due to the cumulative effect of altering multiple such genes at the same time [6]. As cell proliferation is controlled in a tissue-dependent manner, the relative importance of OGs and TSGs varies across tissues, so that the density of tissue-specific driver genes can help predict aneuploidy patterns [7]. Second, some recurrent aneuploidies reflect the chromosome arm-wide gene expression patterns that characterize their normal tissue of origin, suggesting that chromosome-arm gains and losses may ‘hardwire’ pre-existing gene expression patterns [8]. Third, several strong cancer driver genes have been shown to underlie the recurrent aneuploidy of the chromosome-arms on which these genes reside; prominent examples are the tumor suppressors *TP53* and *PTEN*, which have been shown to drive the recurrent loss of chromosome-arm 17p in leukemia and that of 10q in glioma, respectively [9–11]. Fourth, it has been recently proposed that somatic amplifications, including chromosome-arm gains, are positively selected in cancer evolution in order to buffer gene inactivation of haploinsufficient genes in mutation-prone regions [12].

Notably, each previous study focused on a separate aspect of tissue specificity; therefore, the relative contribution of each factor to shaping the overall aneuploidy landscape of human tumors is currently unknown. Furthermore, whether any additional tissue-specific traits could also play a major role in driving aneuploidy patterns remains an open question. Importantly, previous studies focused on the role of positive selection in driving the gain or the loss of specific chromosome-arms in specific tumor types. However, unlike point mutations in specific genes, aneuploidies come with a strong fitness cost [1, 13]. Therefore, whereas positive selection greatly outweighs negative selection in shaping the landscape of point mutations in cancer, as evaluated by a refined version of the normalized ratio of non-synonymous to synonymous mutations [14], both positive selection and negative selection may be important for shaping the landscape of aneuploidy. Indeed, a recent study showed that negative selection could determine the boundaries of recurrent cancer copy number alterations [15]. It is therefore necessary to consider the balance between positive and negative selection in shaping the aneuploidy landscapes of human cancer.

Machine learning (ML) methods have been applied to study a variety of biological and medical questions where heterogeneous large-scale data are available [16]. In the context of cancer, supervised ML methods were applied to predict cancer driver genes [17, 18], to distinguish between cancer types [19, 20], and to predict gene dependency in tumors [21]. However, ML has not been applied to investigate the observed patterns of aneuploidy in human cancer. Whereas ML has been frequently used for prediction and often regarded as a black box, recent advancements have allowed more insight into the factors that underlie prediction. For example, Shapley Additive exPlanations algorithm (SHAP) [22, 23] estimates the importance and relative contribution of each of the features utilized by the model to the model’s decisions.

Here, we present a novel ML approach to elucidate the factors that underlie the cancer type-specific patterns of aneuploidy. For this, we constructed separate ML models for chromosome-arm gain and loss, whereby each of 39 chromosome-arms within 24 cancer types was associated with 20 types of features corresponding to various genomic attributes of chromosome-arms, normal tissues, primary tumors, and cancer cell lines (CCLs). Our approach is focused on interpretation rather than prediction of aneuploidy recurrence patterns. Interpretation of the gain and loss models for aneuploidy in primary tumors captured known genomic features that had been previously reported to shape aneuploidy landscapes, supporting the models’ validity. Furthermore, these analyses suggested that negative selection played a greater role than positive selection in this process and revealed paralog compensation as an important contributor to cancer type-specific aneuploidy patterns, in both primary tumors and CCLs. Lastly, we experimentally validated a specific aneuploidy driver using genetically engineered isogenic human cells.

## Results

### Constructing machine learning models to classify cancer aneuploidy patterns

**Fig. 1:**
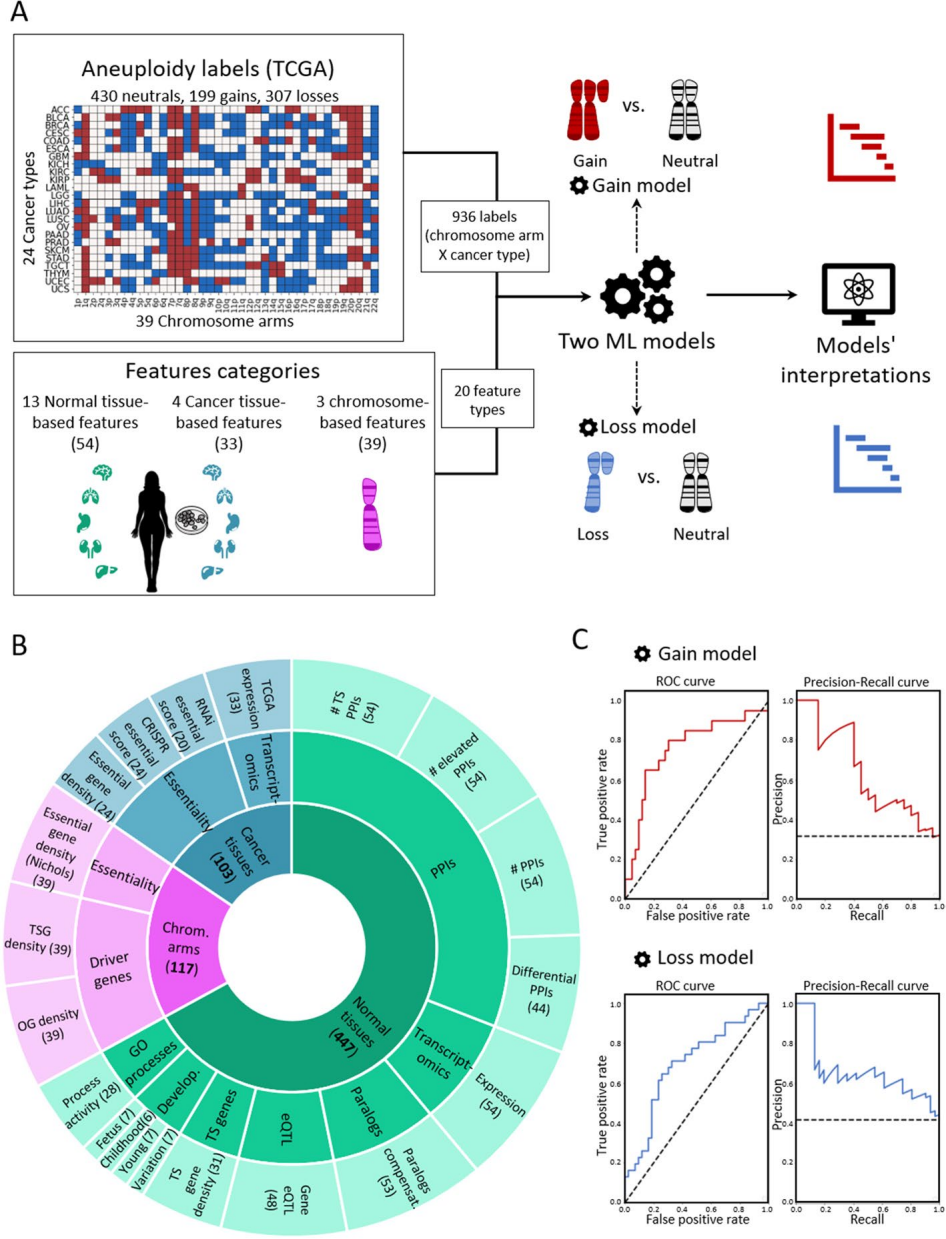
A machine learning (ML) approach for predicting aneuploidy in cancer. **A** Schematic view of the ML model construction. Labels represent aneuploidy status of each chromosome arm in 24 cancer types (abbreviation of cancer types detailed in Additional file 2: Table S1), classified as gained (red, *n* = 199), lost (blue, *n* = 307), or neutral (white, *n* = 430). Features consist of 20 types of features pertaining to chromosome-arms, normal tissues and cancer tissues (see **B**). Two separate ML models were constructed to predict gained and lost chromosome-arms (gain model and loss model). Each model was analyzed to estimate the contribution of the features to the predicted outcome. **B** The features analyzed by the ML model. The inner layer shows feature categories: chromosome arms (purple), cancer tissues (primary tumors and CCLs, blue), and normal tissues (green). The middle layer shows the sub-categories of the features. Chromosome-arm features include essentiality and driver genes features. Cancer-tissue features include transcriptomics and essentiality features. Normal-tissue features include protein–protein interactions (PPIs), transcriptomics, paralogs, eQTL, tissue-specific (TS) genes, development, and GO processes features. The outer layer represents all 20 feature types that were analyzed by the model. Numbers in parentheses indicate the number of tissues, organs, or cell lines from which cancer and normal tissue features were derived, or the number of chromosome-arms from which chromosome-arm features were derived. **C** The performance of the ML models as evaluated by the area under the receiver-operating characteristic curve (auROC, left) and the precision recall curve (auPRC, right) using tenfold cross-validation. Gain model (gradient boosting): auROC = 74% and auPRC = 63% (expected 32%). Loss model (XGBoost): auROC = 70% and auPRC = 63% (expected 42%)

To create a supervised classification ML model that predicts the recurrence pattern of aneuploidy across cancer types, we built a large‐scale dataset consisting of labels and features per instance of chromosome-arm and cancer type. For each instance, the label indicated whether the chromosome-arm was recurrently gained, lost, or remained neutral in that cancer. Labels were determined according to Genomic Identification of Significant Targets in Cancer (GISTIC2.0) [24]. We focused on 24 cancer types for which transcriptomic data of normal tissues of origin was available from the Genotype-Tissue Expression Consortium (GTEx) ([25] (Methods). In total, 199 instances of chromosome-arm and cancer type were labeled as gained, 307 were labeled as lost, and 430 were labeled as neutral (Fig. 1A).

Next, we defined three categories of features (Fig. 1B; Methods). The first category, denoted ‘chromosome-arms’, contained features of chromosome-arms that are independent of cancer type. Chromosome-arm features included the density of OGs, the density of TSGs [6], and the density of essential genes [26] per chromosome-arm. The second category, denoted ‘cancer tissues’, contained features pertaining to chromosome-arms in primary tumors and CCLs. It included features pertaining to expression of genes in primary tumors and essentiality of genes in CCLs. Expression levels of genes in each chromosome-arm per cancer type were obtained from The Cancer Genome Atlas (TCGA, https://www.cancer.gov/tcga). Gene essentiality scores were obtained from the Cancer Dependency Map (DepMap) [27]. In total, this category included 103 omics-based readouts (Methods). The third category, denoted ‘normal tissues’, contained features pertaining to chromosome-arms in normal tissues from which cancer types originated (e.g., colon tissue was matched with colon adenocarcinoma, Additional file 2: Table S1). Features of normal tissues included expression levels of genes located on each chromosome-arm in the respective normal tissue, their tissue protein–protein interactions (PPIs) [28, 29], and their tissue-specific biological process activities [30]. It also included tissue-specific dosage relationships between paralogous genes, denoted ‘paralog compensation’ [31, 32]. In total, this category included 447 tissue-based properties (Methods). To enhance our understanding of cancer and tissue selectivity, feature values of cancer and normal tissues were transformed from absolute to relative; for example, instead of indicating the absolute expression level of a gene in a given normal tissue, the expression feature was set to the expression level of the gene in the given tissue relative to its expression levels in all tissues (Additional file 1: Fig. S1). Each chromosome-arm was then assigned with a feature value that was inferred from the values of its genes (Methods, Additional file 1: Fig. S2).

To fit the features dataset and the labels dataset, we further transformed the features dataset, such that each instance of chromosome-arm and cancer type was associated with features corresponding to the chromosome-arm, cancer type, and matching normal tissue (Methods). In total, the dataset included 20 types of features per chromosome-arm and cancer type: 3 in the chromosome-arm category, 4 in the cancer tissues category, and 13 in the normal tissues category (Fig. 1B). We assessed the similarity between every pair of features using Spearman correlation (Additional file 1: Fig. S3A). Most features did not correlate with each other (Additional file 1: Fig. S3B). Among the correlated feature pairs were PPI-related features and expression in normal adult and developing tissues features (Additional file 1: Fig. S3A). Lastly, we assessed the similarity between instances of chromosome-arm and cancer type by their feature values using principal component analysis (PCA) (Additional file 1: Fig. S3C). Instances did not cluster by their aneuploidy pattern (gain/loss/neutral), suggesting that a more complex model is needed to classify the different patterns.

With these labels and features of each chromosome-arm and cancer type, we set out to construct two separate ML models to predict chromosome-arm gain and loss patterns across cancer types (denoted as the ‘gain model’ and the ‘loss model’, respectively; Fig. 1A). Each model was trained and tested on data of gained (or lost) chromosome-arms versus neutral chromosome-arms. We employed five different ML methods (Methods) and assessed the performance of each method by using tenfold cross-validation and calculating average area under the receiver operating characteristic (auROC) and average area under the precision-recall curve (auPRC) (Additional file 1: Fig. S4A,B). Logistic regression showed similar results to a random prediction, with auROC of 54% for each model (Additional file 1: Fig. S4), indicating that the relationships between features and labels are non-linear. Decision tree methods that can capture such relationships [33, 34], including gradient boosting, XGBoost, and random forest, performed better than logistic regression and similarly to each other (Additional file 1: Fig. S4). Best performance in the gain model was achieved by gradient boosting method, with auROC of 74% and auPRC of 63% (expected: 32%) (Fig. 1C). Best performance in the loss model was achieved by XGBoost, with auROC of 70% and auPRC of 63% (expected: 42%) (Fig. 1C).

### Revealing the top contributors to cancer aneuploidy patterns

The main purpose of our models was to identify the features that contribute the most to the recurrence patterns of aneuploidy observed in human cancer, which could illuminate the factors at play. To this aim, we used the SHAP (Shapley Additive exPlanations) algorithm [22, 23], which estimates the importance and relative contribution of each feature to the model’s decision and ranks them accordingly. We applied SHAP separately to the gain model and to the loss model (Methods).

**Fig. 2:**
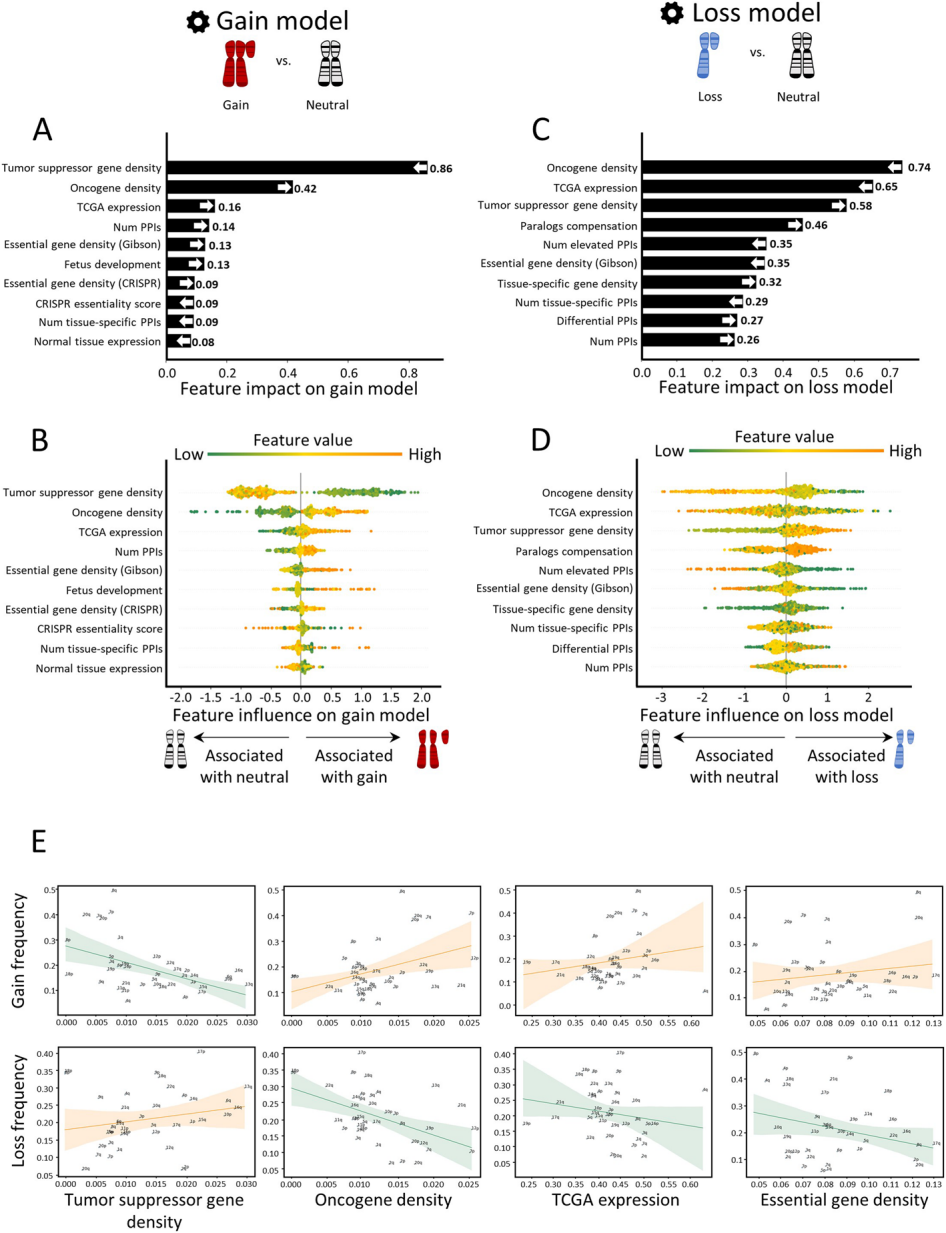
Quantitative views into the ten topmost contributing features of the gain and loss models. Features are ordered from bottom to top by their increased average absolute contribution to the model, as calculated by SHAP. **A** The average absolute contribution of each feature to the gain model. The directionality of the feature (i.e., whether high feature values correspond to gain or neutral) is represented by an arrow. **B** A detailed view of the contribution of each feature to the gain model. Per feature, each dot represents the contribution per instance of a chromosome-arm and cancer type pair. The dots are spread based on whether they were classified as neutral (left) or gain (right) by the model. Instances are colored by the feature value (green-to-orange scale denotes low-to-high value). The order (height) of each feature is the same as in **A**. **C** Same as panel **A** for the loss model. **D** Same as panel **B** for the loss model. **E** The correlations between top contributing features and the frequencies of chromosome-arm gains and losses, as measured by Spearman correlation. *P*-values were adjusted for multiple hypothesis testing using Benjamini–Hochberg procedure. Negative correlation between TSG density and gain frequency (*ρ* = − 0.52, adjusted *p* = 0.006). Positive correlation between TSG density and loss frequency (*ρ* = 0.3, adjusted *p* = 0.17). Positive correlation between OG density and gain frequency (*ρ* = 0.25, adjusted *p* = 0.18). Negative correlation between OG density and loss frequency (*ρ* = − 0.47, adjusted *p* = 0.01). Positive correlation between TCGA expression and gain frequency (*ρ* = 0.29, adjusted *p* = 0.14). Negative correlation between TCGA expression and loss frequency (*ρ* = − 0.33, adjusted *p* = 0.12). Positive correlation between essential gene density and gain frequency (*ρ* = 0.16, adjusted *p* = 0.37). Negative correlation between essential gene density and loss frequency (*ρ* = − 0.1, adjusted *p* = 0.5)

In the gain model, the topmost features were TSG density and OG density (Fig. 2A,B). As expected, these features showed opposite directions: TSG density was low in gained chromosome-arms, whereas OG density was high, in line with previous observations [6, 7] (Fig. 2B). Importantly, this analysis revealed that the impact of TSGs on the gain model’s decision was twice larger than that of OGs (Fig. 2A), highlighting the importance of negative selection for shaping cancer aneuploidy patterns. The third most important feature was TCGA expression, which quantified the expression of arm-residing genes in the given cancer type relative to their expression in other cancers. Notably, expression levels were obtained only from samples where the chromosome-arm was not gained or lost (Methods). This analysis revealed that, across cancer types, chromosome-arms that tend to be gained exhibit higher expression of genes even in neutral cases, consistent with a previous recent study [8]. This confirms that the genes on gained chromosome-arms are preferentially important for the specific cancer types in which these gains are recurrent. Congruently, PPIs and normal tissue expression—features of normal tissues—were also among the ten top-contributing features (Fig. 2A). The estimated importance of all features in the gain model is shown in Additional file 1: Fig. S5A.

The loss model shared the same top three features, yet with opposite directions and different ranks (Fig. 2C,D). OG density ranked first, was low in lost chromosome-arms, whereas TSG density ranked third, was high (Fig. 2D), in line with previous observations [6, 7]. In contrast to the gain model, in the loss model, the impact of OG density on the model’s decision was larger than that of TSG density, again in line with negative selection as an important force in cancer aneuploidy evolution. TCGA expression (computed from samples where the chromosome-arm was not lost or gained, see Methods) ranked second: chromosome-arms with highly-expressed genes tended not to be recurrently lost, in line with negative selection. Another top feature that showed opposite directions between the gain and loss model was essential gene density [26]. As expected, essential gene density was low in lost chromosome-arms, in line with negative selection against losing copies of essential genes [26, 27, 35]. The estimated importance of all features in the loss model is shown in Additional file 1: Fig. S5B.

To examine the direct relationships between high-ranking features and aneuploidy recurrence patterns, we assessed the correlations between these features and aneuploidy prevalence (Methods). In accordance with the SHAP analysis, the negative correlation between TSG density and chromosome-arm gain (*ρ* = − 0.52, adjusted *p* = 0.0006, Spearman correlation; Fig. 2E) was much stronger and more significant than the positive correlation between OG density and chromosome-arm gain (*ρ* = 0.25, adjusted *p* = 0.12, Spearman correlation; Fig. 2E). Similarly, the negative correlation between OG density and chromosome-arm loss (*ρ* = − 0.47, adjusted *p* = 0.003, Spearman correlation; Fig. 2E) was much stronger and more significant than the positive correlation between TSG density and chromosome-arm loss (*ρ* = 0.3, adjusted *p* = 0.067, Spearman correlation; Fig. 2E). TCGA expression and essential gene density were correlated with chromosome-arm gain, and anticorrelated with chromosome-arm loss, albeit to a lesser extent (Fig. 2E, Additional file 1: Fig. S6). Also showing positive correlations with gains and negative correlations with losses were features derived from expression levels in normal adult and developing tissues, certain PPI-related features, and additional essentiality features (Additional file 1: Fig. S6). However, these correlations were weaker than the correlations described above. Altogether, correlation analyses supported the relationships between top features of each model and aneuploidy patterns.

### The robust impact of top contributors to cancer aneuploidy patterns

Next, we asked if the above results were sensitive to our model construction schemes. We first tested the robustness of the models to internal parameters used to generate the features (Methods). We therefore recreated features upon modifying internal parameters and repeated model construction and interpretation (Methods). We found that feature importance was robust to these changes (Additional file 1: Fig. S7, Additional file 3: Table S2). Second, we tested the robustness of the results upon tuning the hyperparameters of each model (Methods, Additional file 1: Fig. S8). The top contributing features of each model were retained following hyperparameter tuning, supporting their reliability (Additional file 1: Fig. S8C). We also checked whether the same top features would be recognized upon modeling one type of chromosome-arm event versus all other events. Applying the same approaches, we constructed two additional ML models. One model classified chromosome-arm gain versus no-gain (i.e., chromosome-arm loss or neutrality). Another model classified chromosome-arm loss versus no-loss (i.e., chromosome-arm gain or neutral). These additional models performed similarly to their respective models (Additional file 1: Fig. S9). SHAP analysis of the two additional models revealed that feature importance was very similar between these models and the original models, which compared gained and lost chromosome-arms only to neutral chromosome-arms (Additional file 1: Fig. S9).

We next tested whether the results were driven by a small subset of chromosome-arm and cancer type instances. For that, per model, we identified chromosome-arm and cancer type instances with the top contributions to the five topmost important features (Methods, Additional file 4: Table S3A,B, Additional file 5: Table S4A,B). Most instances contributed to at least one of these features, and none of the instances contributed to all five (Additional file 5: Table S4C). Next, we focused on chromosome-arm and cancer type instances that were top contributors to at least three of the five features (4.3% and 1.9% of the pairs in the gain and loss models, respectively). We tested their impact on the model by excluding them from the dataset and repeating the construction and interpretation of each model without them. The revised gain model retained its five topmost important features, though their ranking slightly changed (the third and fifth features switched). The revised loss model retained its four topmost important features (the fifth and seventh features switched) (Additional file 1: Fig. S10). This suggests that the general effect of the features was not driven by a small subset of instances.

Lastly, we expanded our analyses to address whole-chromosome gains and losses. For this, we updated the features dataset to refer to whole-chromosome and cancer type instances (Methods). For example, the feature TSG density was updated to refer to the entire chromosome. Likewise, we updated the aneuploidy status of whole-chromosome and cancer type instances using data from GISTIC (Methods). This resulted in a dataset of 78 whole-chromosome gains, 151 whole-chromosome loss, and 299 neutral cases. Next, we used these data to train a whole-chromosome gain (trisomy) model and a whole-chromosome loss (monosomy) model. Model training and assessment were similar to the chromosome-arm gain and loss models. Specifically, we employed five different ML methods and assessed their performance using fivefold cross-validation. Best performance for the trisomy model was achieved by random forest, with auROC of 69% and auPRC of 47% (expected 21%; Additional file 1: Fig. S11A). Best performance for the monosomy model was achieved by XGBoost, with auROC of 71% and auPRC of 59% (expected 34%; Additional file 1: Fig. S11D). Performances were somewhat weaker than the chromosome-arm models, in accordance with the training data being almost twofold smaller. Lastly, we interpreted each model using SHAP. In the trisomy model, the topmost feature was TSG density and its impact was over twofold larger than the impact of other features, similarly to the chromosome-arm gain model (Additional file 1: Fig. S11B,C). Other strong features of the chromosome-arm gain model, TCGA expression and OG density, ranked fifth and sixth, yet preserved their directionality. In the monosomy model, top features included OG density, TCGA expression, and paralogs compensation, fitting with the chromosome-arm loss model (Additional file 1: Fig. S11E,F). The feature TSG density was ranked eight, yet preserved its directionality, similarly to the remaining features. Altogether, these results suggest that negative selection is an important factor in shaping both chromosome-arm and whole-chromosome aneuploidy patterns.

### Similar features shape aneuploidy patterns in human cancer cell lines and in human tumors

**Fig. 3:**
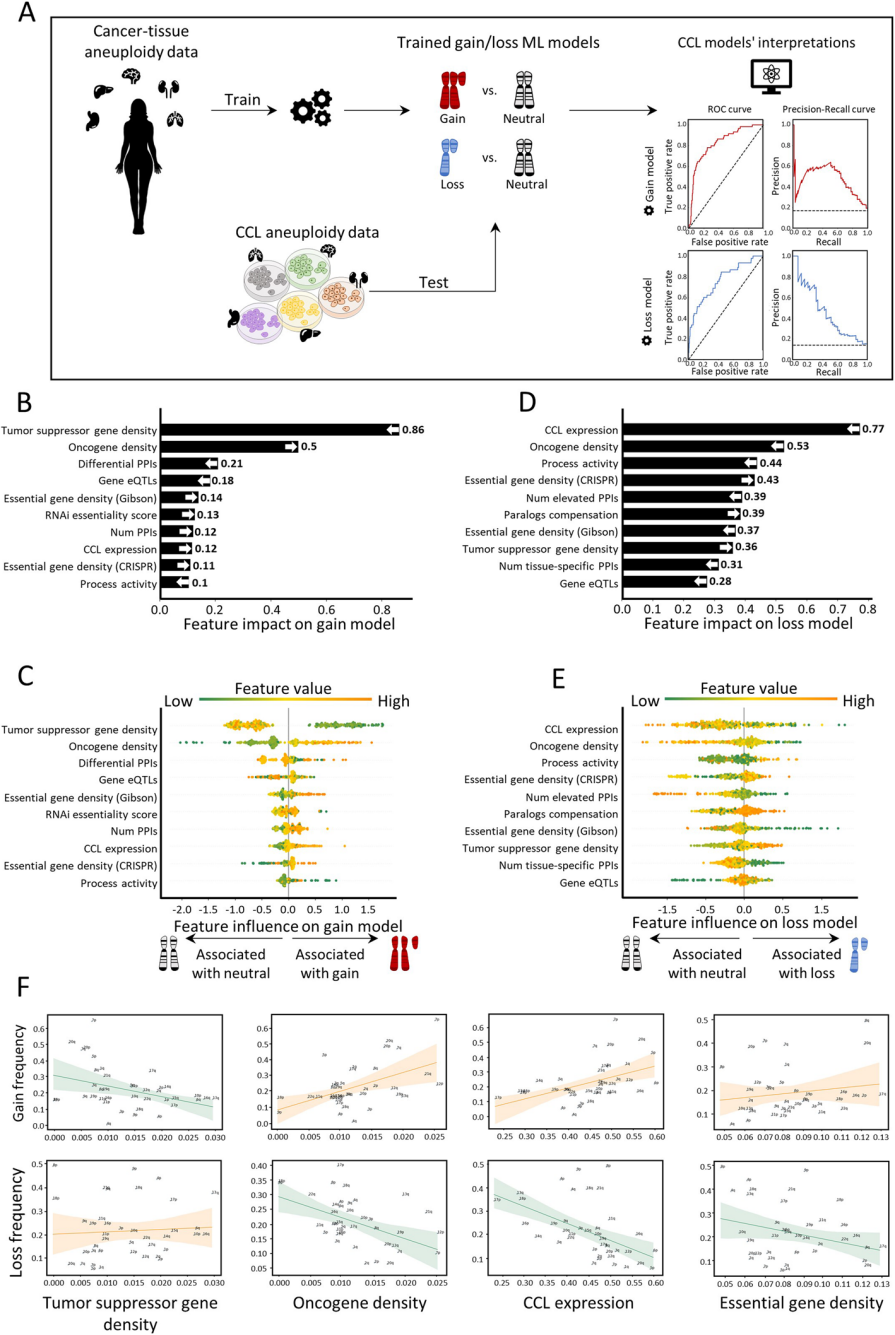
Aneuploidy patterns in CCLs and primary tumors are shaped by similar features. **A** The ML scheme for analysis of aneuploidy patterns in CCLs. The gain and loss models that were trained on aneuploidy patterns in primary tumors were applied to aneuploidy patterns in CCLs. Performance was measured using tenfold cross-validation. Gain model (gradient boosting): auROC = 83%, auPRC = 49% (expected 15%). Loss model (XGBoost): auROC = 76%, auPRC = 45% (expected 11%). **B** The average absolute contribution of the ten topmost features to the gain model (see legend of Fig. 2A). The order and directionality of the features generally agree with the gain model in primary tumors. **C** A detailed view of the contribution of the ten topmost features to the gain model (see legend of Fig. 2B). **D** Same as **B** for the loss model. The order and directionality of the features generally agree with the loss model in primary tumors. **E** Same as panel **C** for the loss model. **F** The correlations between top contributing features and the frequencies of chromosome-arm gains and losses, as measured by Spearman correlation. *p*-values were adjusted for multiple hypothesis testing using Benjamini–Hochberg procedure. Negative correlation between TSG density and gain frequency (*ρ* = − 0.37, adjusted *p* = 0.04). Positive correlation between TSG density and loss frequency (*ρ* = 0.17, adjusted *p* = 0.32). Positive correlation between OG density and gain frequency (*ρ* = 0.44, adjusted *p* = 0.012). Negative correlation between OG density and loss frequency (*ρ* = − 0.28, adjusted *p* = 0.13). Positive correlation between CCL expression and gain frequency (*ρ* = 0.53, adjusted *p* = 0.002). Negative correlation between CCL expression and loss frequency (*ρ* = − 0.6, adjusted *p* = 0.0006). Positive correlation between essential gene density and gain frequency (*ρ* = 0.18, adjusted *p* = 0.33). Negative correlation between essential gene density and loss frequency (*ρ* = − 0.17, adjusted *p* = 0.32)

Next, we aimed to test whether similar features also shape aneuploidy patterns in CCLs. We collected data of aneuploidy patterns of all chromosome-arms in CCLs [36] and analyzed 10 cancer types with matched normal tissue data from GTEx [25] (Methods). Similar to the analysis of cancer tissues, we labeled each instance of chromosome-arm and CCL as recurrently gained (59 instances), recurrently lost (45 instances), or neutral (286 instances) and updated the features associated with cancer types according to the CCL data (Methods). We then applied the gain and loss ML models, which were trained on primary tumor data, to identify determinants of aneuploidy patterns of CCLs (Methods). The performance of the models was at least as good as for primary tumors (gain model: auROC = 83% and auPRC = 49% (expected 15%); loss model: auROC = 76% and auPRC = 45% (expected 11%), Fig. 3A). These results indicate that similar factors affect aneuploidy in cancers and in CCLs, consistent with the highly similar aneuploidy patterns observed in tumors and in CCLs [36, 37].

We next used SHAP to assess the contribution of each feature to each of the models. TSG density and OG density remained the top contributing features for the gain model. Consistent with our results in primary tumors, the contribution of TSG density was much stronger than that of OG density, confirming the role of negative selection (Fig. 3B,C). In the loss model, the ranking of top features was slightly different than in primary tumors (Fig. 3D). Expression in CCL was the top feature, such that recurrently lost chromosome-arms were associated with lower gene expression in neutral cases. OG density was one of the strongest contributing features for the loss model whereas TSG density had weaker contribution, again in line with negative selection playing an important role in shaping cancer aneuploidy landscapes (Fig. 3D,E). Certain features of normal tissues were also highly ranked. The contribution of essential gene density was also consistent with its impact in primary tumors (Fig. 3B,C).

As with the primary tumors, correlation analyses supported the contributions of the different features. CCL expression was highly correlated with chromosome-arm gain and anticorrelated with chromosome-arm loss (*ρ* = 0.54, adjusted *p* = 0.02, and *ρ* = − 0.6, adjusted *p* = 0.0006, respectively; Fig. 3F). Negative correlations were also observed between TSG density and gain frequency (*ρ* = − 0.37, adjusted *p* = 0.04, Spearman correlation; Fig. 3F) and between OG density and loss frequency (*ρ* = − 0.28, adjusted *p* = 0.1, Spearman correlation; Fig. 3F). Altogether, these results indicate that despite the continuous evolution of aneuploidy throughout CCL culture propagation [38], similar features drive aneuploidy recurrence patterns in primary tumors and in CCLs.

### Chromosome 13q aneuploidy patterns are tissue-specific, and KLF5 is a driver of 13q gain in colorectal cancer

**Fig. 4:**
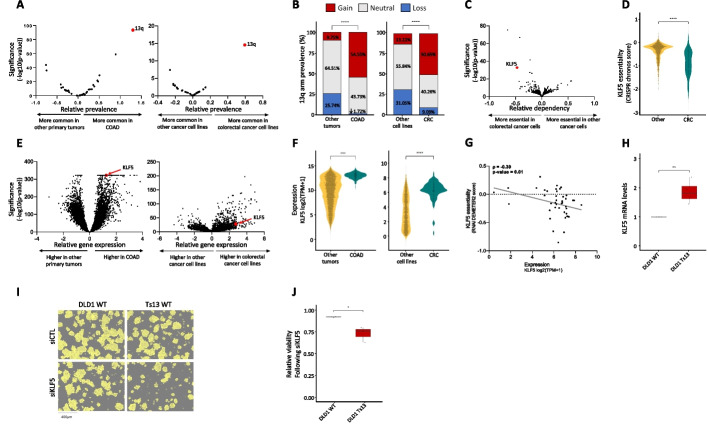
*KLF5* is a potential driver of chromosome 13q gain in human colorectal cancer. **A** Comparison of the prevalence of chromosome-arm aneuploidies in colorectal tumors against all other tumors (left) and colorectal cancer cell lines against all other cancer cell lines (right). On the right side are the aneuploidies that are more common in colorectal cancer, and on the left side are the ones that are less common in colorectal cancer. Chromosome-arm 13q (in red) is the top differential aneuploidy in colorectal cancer. **B** Comparison of the prevalence of 13q aneuploidy between colorectal tumors and all other tumors (left) and between colorectal cancer cell lines and all other cancer cell lines (right). ****, *p* < 0.0001 and ****, *p* < 0.0001; Chi-square test. **C** Genome-wide comparison of differentially essential genes between colorectal cancer cell lines (*n* = 85) and all other cancer cell lines (*n* = 1407). On the right side are the genes that are more essential in other cancer cell lines, and on the left side are those that are more essential in colorectal cancer, based on a genome-wide CRISPR/Cas9 knockout screens [39]. The *x*-axis presents the effect size (i.e., the differential response between colorectal cell lines and other cell lines), and the *y*-axis presents the significance of the difference (-log10(*p*-value)). *KLF5* (in red) is the second most differentially essential gene in colorectal cancer cell lines. **D** Comparison of the sensitivity to CRISPR knockout of *KLF5* between colorectal cancer cell lines (*n* = 59) and all other cancer cell lines (*n* = 1041). ****, *p* < 0.0001; two-tailed Mann–Whitney test. **E** Genome-wide comparison of differentially expressed genes between colorectal tumors (*n* = 434) and all other tumors (on the left, *n* = 11,060) and between colorectal cancer cell lines (*n* = 85) and all other cancer cell lines (on the right, *n* = 1407). On the right side are the genes that are over-expressed in colorectal cancer and on the left side are those that are over-expressed in other cell lines. *KLF5* (in red) significantly over-expressed in colorectal cancer. **F** Comparison of *KLF5* mRNA levels between colorectal tumors (*n* = 434) and all other tumors on the left (*n* = 11,060) and between colorectal cancer cell lines (*n* = 85) and all other cancer cell lines (on the right, *n* = 1407). ****, *p* < 0.0001; two-tailed Mann–Whitney test. **G** Correlation between *KLF5* mRNA expression and the sensitivity to *KLF5* knockdown, showing that higher *KLF5* expression is associated with increased sensitivity to its RNAi-mediated knockdown. *ρ* = − 0.39, *p* = 0.01; Spearman correlation. **H** Comparison of *KLF5* mRNA levels between DLD1-WT (without trisomy of chromosome 13) and DLD1-Ts13 (with trisomy of chromosome 13) colorectal cancer cells. **, *p* = 0.0025; one-sample *t*-test. **I** Representative images of DLD1-WT and DLD1-Ts13 cells treated with siRNA against *KLF5*. DLD1-Ts13 cells proliferated more slowly, as previously reported, but were more sensitive to the knockdown after accounting for their basal proliferation rate. Cell masking (shown in yellow) was performed using live cell imaging (IncuCyte) following 72 h of treatment. Scale bar 400µm. **J** Quantification of the relative response to *KLF5* knockdown between DLD1-WT and DLD1-Ts13, as evaluated by quantifying cell viability in cells treated with siRNA against *KLF5* versus a control siRNA for 72 h. *n* = 3 independent experiments. *, *p* = 0.0346; one-sided paired *t*-test

In human cancer, a chromosome-arm is either recurrently gained across cancer types or it is recurrently lost across cancer types, but rarely is a chromosome-arm both gained in some cancer types and lost in others [4, 5]. An intriguing exception is chr13q. Of all chromosome-arms, chr13q is the chromosome-arm with the highest density of tumor suppressor genes (Fig. 2E). It is therefore not surprising that chr13q is recurrently lost across multiple cancer types (with a median of 30% of the tumors losing one copy of 13q across cancer types) [4, 5]. Interestingly, however, chr13q is recurrently gained in human colorectal cancer (in 58% of the samples), suggesting that it can confer a selection advantage to colorectal cells in a tissue-specific manner. Indeed, when comparing colorectal tumors and colorectal cancer cell lines against all other cancer types, chr13q was the top differentially affected chromosome-arm (Fig. 4A,B). We therefore set out to study the basis for this unique tissue-specific aneuploidy pattern.

We performed a genome-wide comparison of differentially essential genes between colorectal cell lines and all other cell lines. The two top genes, which are much more essential in colorectal cancer cells than in other cancer types, were CTNNB1 and *KLF5* (Fig. 4C). Of particular interest is *KLF5*, which is located on chr13q and colorectal cancer cell lines are significantly more sensitive to its knockout (Fig. 4D). *KLF5* was reported to be tumor-suppressive in the context of several cancer types, such as breast and prostate [40, 41]. In colon cancer, however, not only is *KLF5* important for tissue identity [42], but it was also reported to be haploinsufficient [43], potentially explaining why loss of chr13q is so rare in colorectal cancer. In line with a potential driving role in the recurrence of chr13q gain in colorectal cancer, *KLF5* was among the most significantly overexpressed genes in colorectal tumors and in colorectal cell lines versus all other cancer types (Fig. 4E,F). Furthermore, *KLF5* expression levels correlated with the cells’ sensitivity to its knockdown (Fig. 4G). To confirm the association between chr13q gain and *KLF5* expression and dependency, we next turned to an isogenic system of human colon cancer cells (DLD1) into which trisomy 13 had been introduced (DLD1-Ts13) [44]. Using this unique experimental system, we confirmed that trisomy 13 results in overexpression of *KLF5* (Fig. 4H) and increased sensitivity to its siRNA-mediated genetic depletion (Fig. 4I,J and Additional file 1: Fig. S12, Additional file 1: Fig. S13). This differential response was specific to *KLF5*, as the trisomy did not affect the sensitivity of the cells to a control siRNA (Additional file 1: Fig. S14), to knockdown of an unrelated gene residing on chr13q (*NEK3*; Additional file 1: Fig. S15), or to knockdown of another transcription factor that plays a role in colon development and is located on another chromosome (*TTC7A*, located on chr2p; Additional file 1: Fig. S16). We, therefore, propose that *KLF5* contributes to the uniquely variable pattern of chr13q aneuploidy across cancer types.

### Paralog compensation is an important feature shaping tissue-specific aneuploidy patterns

One of the topmost contributing features to the chromosome-arm loss model in primary tumors and in CCLs, as well as to the whole-chromosome loss model, was paralog compensation. It was previously shown that while loss of genes with paralogs was less detrimental than loss of singleton genes [45], the impact of gene loss in a specific condition depends on the expression level of its paralog [46]. The paralog compensation feature was therefore designed to quantify the expression ratio between two paralogs. Specifically, higher values of this feature for a given gene correspond to a higher expression of the paralog relative to the gene (Methods). Previous studies of hereditary disease genes showed that lower paralog compensation in a tissue was associated with disease manifestation in that tissue [31, 32]. Paralog compensation was also shown in cancer tissues: In CCLs, essentiality of a gene was decreased with an increased expression of its paralog [27, 46, 47]. In primary tumors, paralog compensation was shown to be associated with increased prevalence of non-synonymous mutations [48] and to correlate with the prevalence of homozygous gene deletion [49]. However, the contribution of paralog compensation to aneuploidy has not been studied to date.

**Fig. 5:**
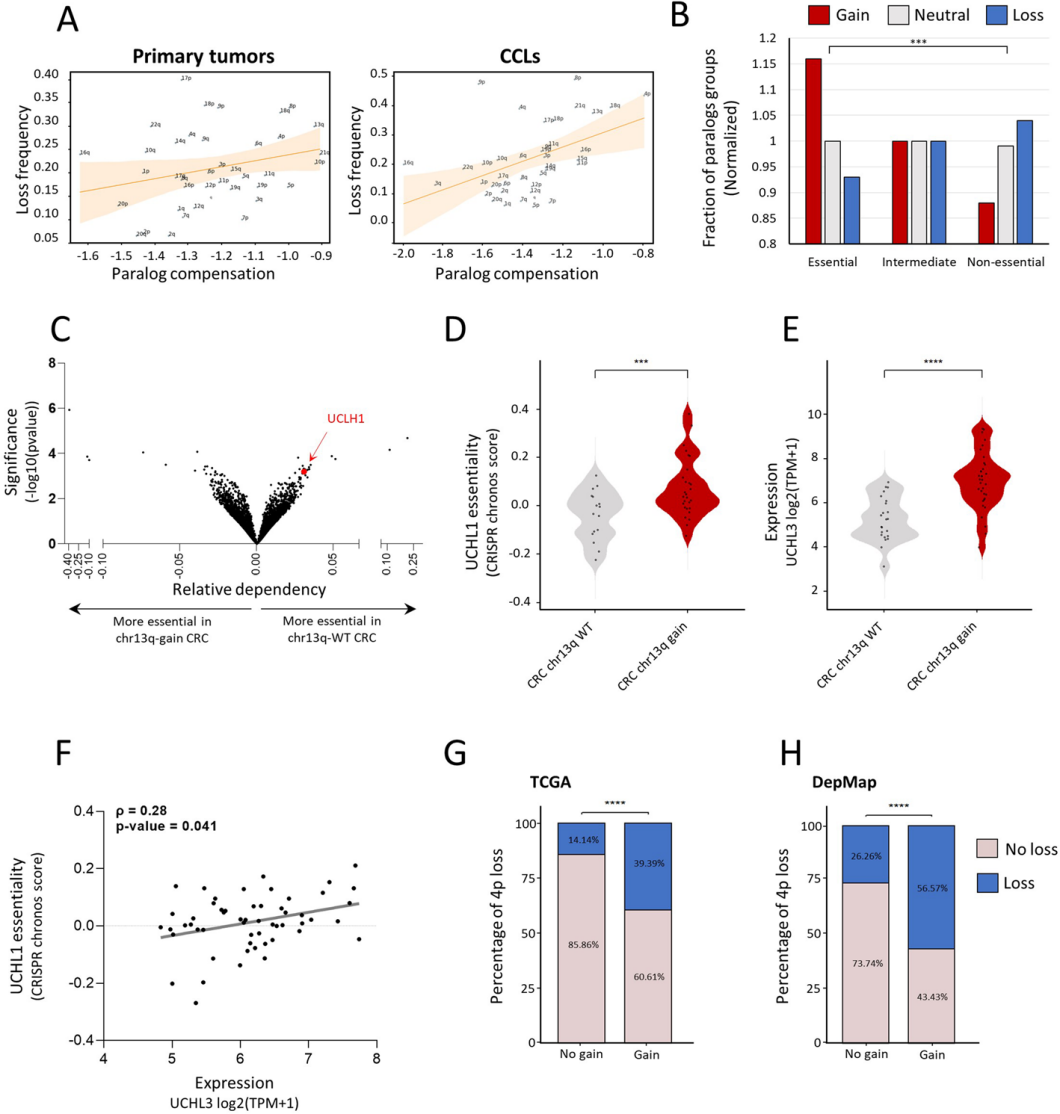
Paralog compensation is an important feature shaping tissue-specific aneuploidy patterns. **A** The correlation between paralog compensation values and loss frequency of chromosome arms in primary tumors (left, *ρ* = 0.26, adjusted *p* = 0.18, Spearman correlation) and in CCLs (right, *ρ* = 0.46, adjusted *p* = 0.01, Spearman correlation). **B** A view into the aneuploidy patterns of paralogs of recurrently lost genes. Recurrently lost genes were divided into essential, intermediate, and non-essential groups. Paralogs of essential genes were more frequently gained, whereas paralogs of non-essential genes were more frequently lost. **C** Genome-wide comparison of differentially essential genes in colorectal cell lines with chr13q gain (*n* = 39) versus chr13q-WT colorectal cell lines (*n* = 25). On the right side are the genes that are more essential in chr13q-WT cells, and on the left side those that are more essential in chr13q-gain cells, based on a genome-wide CRISPR/Cas9 knockout screens [39]. The *x*-axis presents the effect size (i.e., the differential response between chr1q-WT and chr13q-gain colorectal cell lines) and the *y*-axis presents the significance of the difference (-log10(p-value)). *UCHL1* (in red) is one of the top genes identified to be more essential in chr13q-WT cells. **D** Comparison of the sensitivity to CRISPR knockout of *UCHL1* between colorectal cell lines with (*n* = 28) and without chr13q gain (*n* = 16). ***, *p* = 0.0003; two-tailed Mann–Whitney test. **E** Comparison of *UCHL3* mRNA expression between colorectal cell lines with (*n* = 34) and without chr13q gain (*n* = 23). ****, *p* < 0.0001; two-tailed Mann–Whitney test. **F** Correlation between *UCHL3* mRNA expression and the sensitivity to *UCHL1* knockout, showing that higher *UCHL3* mRNA levels are associated with reduced sensitivity to *UCHL1* knockout. *ρ* = 0.28, *p* = 0.041; Spearman correlation. **G** Comparison of the prevalence of chr4p loss between human primary colorectal tumors with and without chr13q gain. ****, *p* < 0.0001, Chi-square test. **H** Comparison of the prevalence of chr4p loss between human colorectal cancer cell lines with and without chr13q gain. ****, *p* < 0.0001, Chi-square test

Paralog compensation ranked fourth and sixth in the loss models of primary tumors and CCLs, respectively (Fig. 2C, Fig. 3D). In both, chromosome-arm loss was associated with higher paralog compensation, suggesting that loss is facilitated by higher relative expression of paralogs (Fig. 2D, Fig. 3E). We also analyzed the correlations between the frequency of chromosome-arm loss and paralog compensation (Methods, Fig. 5A). Indeed, the frequency of chromosome-arm loss was positively correlated with paralog compensation in both primary tumors and in CCLs (*ρ* = 0.26 and *ρ* = 0.46, respectively, Spearman correlation; Fig. 5A).

Next, we tested whether paralog compensation, namely gain or overexpression of paralogs, could indeed facilitate chromosome-arm loss. We started by grouping genes in recurrently lost chromosome-arms into essential, intermediate, or non-essential, according to their essentiality in CCLs [27] (Methods). We then associated each gene with the aneuploidy status of the chromosome-arm of its paralog, namely whether the chromosome-arm of the paralog was gained, lost, or remained neutral in the corresponding CCL (Methods, Additional file 1: Fig. S17A). The fraction of genes with paralogs on neutral chromosome-arms was similar in all essentiality groups (Fig. 5B). In contrast, the fraction of gained paralogs was highest in the group of essential genes and lowest in the group of non-essential genes. This suggests that the loss of essential genes is more likely accompanied by the gain of their paralogs. Likewise, the fraction of lost paralogs was lowest in the group of essential genes and highest in the group of non-essential genes (*p* = 2.38e − 24, Chi-square test; Fig. 5B). This suggests that the loss of essential genes is less likely to be accompanied by the loss of their paralog. The same trend was shown upon comparing the distribution of essentiality scores between genes with gained paralogs versus genes with lost paralogs (*p* = 9.2e − 16, KS test; Additional file 1: Fig. S17B). Hence, paralog compensation can facilitate chromosome-arm loss.

Next, we decided to identify a specific example. In human colon cancer, the long arm of chromosome 13 (chr13q) is commonly gained, as described above, whereas the short arm of chromosome 4 (chr4p) is commonly lost [5, 37]. We analyzed the association between chr13q-residing genes and the essentiality of their paralogs, revealing *UCHL3* (chr13q)-*UCHL1* (chr 4p) as the most significant correlation (Additional file 6: Table S5 and Fig. 5C). Human colon cancer cell lines with chr13q gain were less sensitive to CRISPR/Cas9-mediated knockout of *UCHL1* (Fig. 5D). Consistently, chr13q-gained cell lines had significantly higher mRNA levels of *UCHL3* (Fig. 5E), and the expression of *UCHL3* was significantly correlated with the essentiality of *UCHL1* (Fig. 5F). We hypothesized that the relationship between these paralogs may affect the co-occurrence patterns of the chromosome-arms on which they reside. Indeed, both in primary human colon cancer and in colon cancer cell lines, loss of chr4p was significantly more prevalent when chr13q was gained (Fig. 5G,H). Together, these results demonstrate that paralog compensation can be affected by—and contribute to the shaping of—aneuploidy patterns.

## Discussion

Recurrent aneuploidy patterns are an intriguing phenomenon that is only partly understood. Several previous studies characterized the unique patterns of aneuploidy in cancer [4, 5, 50] or attempted to identify the driving role of a specific aberration in a specific cancer context [9, 51–54]. Attempts to explain copy number patterns in cancer focused on specific pre-defined aspects, such as the specific boundaries of the alterations [15], the densities of OGs and TSGs on the aberrant chromosomes [6, 7] or the gene expression changes that they induce [8], and these aspects were interrogated using statistical methods and correlation analyses. Here, in contrast, we studied this phenomenon using an unbiased ML-based approach. As with other ML applications, it allowed us to study multiple aspects simultaneously. Yet, unlike classical ML-based studies that mainly aim to improve prediction, for example by using deep learning to predict gene dependency in tumors [21], our focus was on interpretability. In fact, we built chromosome-arm gains and loss models only to then identify factors that shape aneuploidy patterns. Interpretable ML was recently applied to reveal genetic attributes that contribute to the manifestation of Mendelian diseases [55]. In this study, we applied interpretable ML for the first time in the context of aneuploidy and at chromosome-arm resolution.

The capability of ML to concurrently assess multiple features opened the door for assessing the relevance of features that have not been rigorously studied to date, such as paralog compensation. Yet, ML has its limitations. Mainly, the number of features that could be analyzed depends on the size of the labeled dataset [56], which, in aneuploidy, was restricted by the number of chromosome-arms and cancer types. We therefore analyzed 20 types of features and tested linear regression and tree-based ML methods, which, unlike deep learning, are suitable for this size of data. Following prediction, our main goal was to assess the relative contribution of each feature to the model’s decision and its directionality using SHAP. Nevertheless, SHAP results should be interpreted with caution. First, SHAP assumes feature independence, although features could be correlated with each other or confounded. Importantly, we found that only a small subset of features correlated with each other, and they did not include the topmost contributing features (Additional file 1: Fig. S3A). Second, the top contributing factors could be correlated with prediction strength, rather than being causal. Lastly, due to the hierarchical nature of decision trees, features that are located low in the decision tree explain only a small fraction of the cases. To estimate feature contribution and directionality more broadly, we explicitly correlated feature values with chromosome-arm gain and loss frequency, finding support for their broad relevance (Fig. 2E, Additional file 1: Fig. S6). We also conducted multiple analyses that tested the robustness of the results to the models’ construction schemes (Additional file 1: Fig. S7, S8), the modeled events (one event versus rest, Additional file 1: Fig. S9; whole-chromosome, Additional file 1: Fig. S11), or to a subset of the chromosome-arm and cancer type instances (Additional file 1: Fig. S10). The different analyses repeatedly revealed the same factors at play, supporting the reliability of our results.

The features that we studied included known and previously underexplored attributes of chromosome-arms, healthy tissues and cancer cells (Fig. 1A,B). OG and TSG densities, which have previously been observed to be enriched on gained and lost chromosome-arms, respectively [6, 7], were top contributing features in both models, thereby supporting the validity of our approach (Fig. 2A,C). In the gain model in particular, their contribution was over 2.6 and 5 times stronger, respectively, than any other feature (Fig. 2A). As our TSG and OG features were cancer-independent, their importance may explain the observation that certain chromosome-arms tend to be either gained or lost across multiple cancer types [4, 5]. Their relative contribution, however, was surprising. In both models, negative associations were much stronger than positive associations: OG density contributed to chromosome-arm loss more than TSG density, implying that it was more important to maintain OGs than to lose TSG (Fig. 2B,D). The reciprocal relationship was true for chromosome-arm gain, as it was more important to maintain TSGs than to gain OGs (Fig. 2A,C). These results were validated using correlation analyses (Fig. 2E) and were recapitulated in CCLs (Fig. 3) and in the analysis of whole-chromosome gains and losses (Additional file 1: Fig. S11). Together, they highlight the importance of negative selection for shaping cancer aneuploidy landscapes [1, 15].

A known factor that contributed to both models was gene expression in primary tumors (TCGA expression, Fig. 2) and in CCLs (CCL expression, Fig. 3). This result suggests that cancers tend to gain chromosome-arms that are enriched for highly-expressed genes and tend to lose chromosome-arms that are enriched for lowly expressed genes. A Similar trend was shown recently for gene expression in normal tissues [8]. Our approach was capable of comparing the relative contributions of both features. We found that the contribution of gene expression in normal tissue was lower than that in cancer tissues, as also evident by its lower correlation with the frequencies of chromosome-arm gains and losses (Additional file 1: Fig. S6). Nevertheless, other features that were derived from gene expression in normal tissues ranked highly, such as the number of PPIs in the gain model and paralog compensation in the loss model, and hence expression in normal tissues is also important (Fig. 2).

A previously under-explored feature that we considered was paralog compensation. Paralog compensation was shown to play a role in the manifestation of Mendelian and complex diseases [31, 32] and in the dispensability of genes in tumors [48, 49] and CCLs [27, 46, 47], but was not studied in the context of aneuploidy. Here, paralog compensation was among the top contributors to the loss model (Fig. 2C, Fig. 3D). The directionality of this feature and correlation analyses showed that, relative to genes located on neutral chromosome-arms, genes located on lost chromosome-arms tend to have higher compensation by paralogs (Fig. 5A). This suggests that chromosome-arm loss is facilitated, or better tolerated, through paralogs’ expression. We also showed that the more essential recurrently lost genes are, the more likely they are to be associated with gains of paralog-bearing chromosome-arms (Fig. 5B). We further demonstrated this for a specific example (the *UCHL3*-*UCHL1* paralog pair; Fig. 5). Overall, our analysis reveals that compensation between paralogs through expression or chromosome-arm gain plays an important role in shaping the landscape of chromosome-arm loss.

**Fig. 6:**
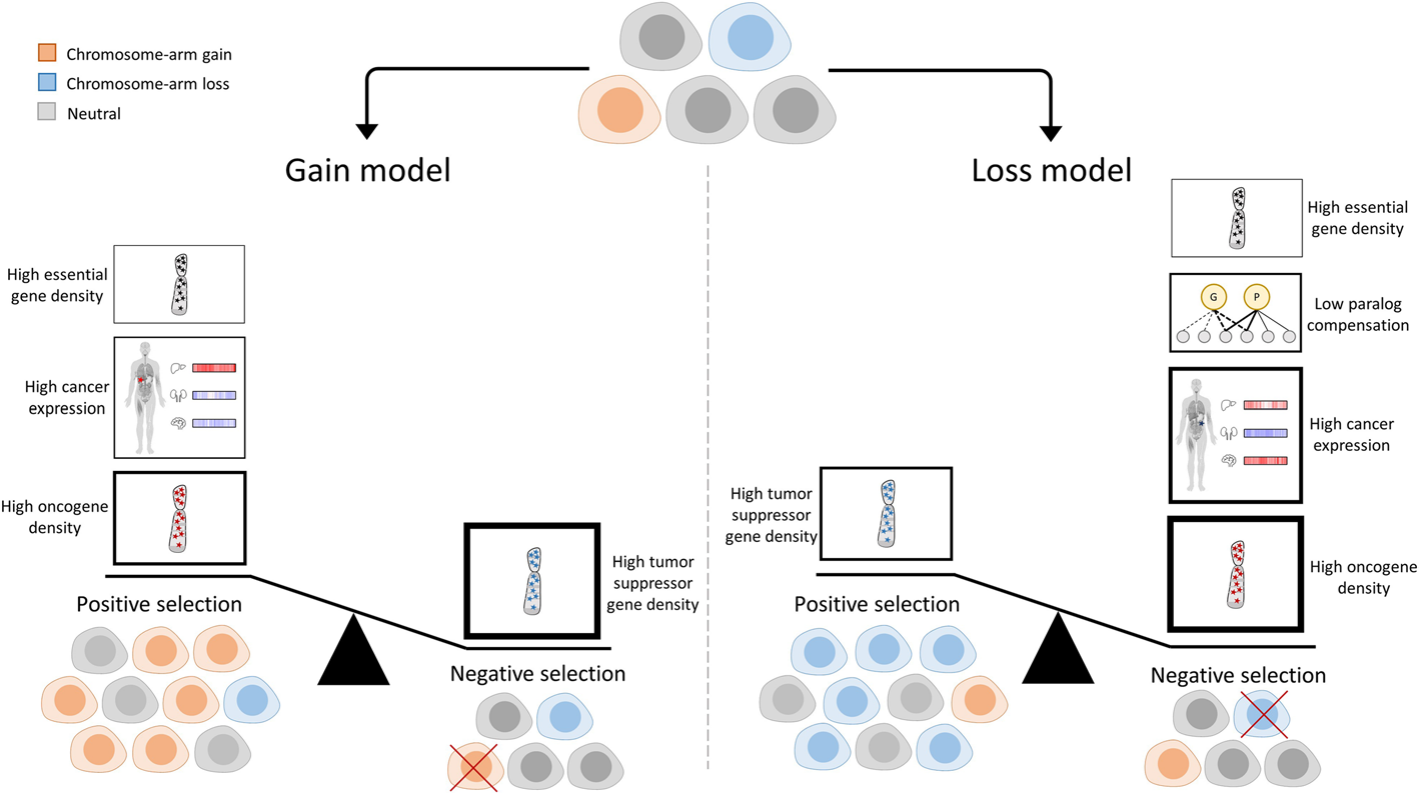
A schematic presentation of the results of the study. Cancer evolution is shaped by negative and positive selection leading to enrichment or depletion of cells with distinct aneuploidy patterns. In the gain model (left), main contributors to positive selection of gained chromosome arms are: (1) high oncogene density, (2) high expression of genes in the cancer tissue, and (3) high essential gene density. A major contributor to negative selection is high tumor suppressor gene density. Importantly, the density of TSGs is more important than the density of OGs for predicting chromosome-arm gains. In the loss model (right), a main contributor to positive selection of lost chromosome arms is high tumor suppressor gene density. Major contributors to negative selection are high oncogene density, high expression of genes in the cancer tissue, low compensation by paralogs, and high density of essential genes. In both models, the features associated with negative selection have higher overall contribution than features associated with positive selection. The thickness of the borders of the boxes reflects the relative contribution of the features to the model

Combining the different results, our models reveal a previously under-appreciated role for negative selection in driving human cancer aneuploidy. This was evident by the tendency not to lose chromosome arms with high OG density, high frequency of essential genes, or low compensation by paralogs, and not to gain chromosome arms with high TSG density (Fig. 6). Previous studies have shown that positive selection outweighs negative selection in shaping the point mutation landscape of human tumors [14]. However, the strong fitness cost associated with aneuploidy suggests that the aneuploidy landscape of tumors might be strongly affected by negative selection as well (reviewed in [1]). Interestingly, evidence for the involvement of negative selection in shaping the copy number alteration (CNA) landscapes of tumors has been proposed in a recent study that analyzed CNA length distributions across human tumors [15]. Our study thus lends further independent support to the importance of negative selection in shaping the landscape of aneuploidy across human cancers (Fig. 6).

Our genome-wide analysis could be expanded in future studies in several ways: (1) While we focused on the top-contributing features, other features, such as PPIs that contributed to both gain and loss models, are also relevant and remain to be studied in depth. (2) It will be interesting to consider additional types of aneuploidy, such as tetrasomies, and explore how whole-genome doubling affects the importance of the features in shaping the aneuploidy landscapes of tumors. (3) Tumors often exhibit heterogeneous (mosaic) aneuploidy patterns [57–60]. Our analyses were entirely based on bulk-population data, and our results therefore describe the selection pressures that shape the landscape of clonal aneuploidies. As more single-cell omics data becomes available, it will be interesting to also study the selection pressures that shape subclonal aneuploidy patterns. (4) Aneuploidies do not always arise independently, so that chromosome-arm events can co-occur or be mutually exclusive [37]. We show that only a small fraction of chromosome-arm events co-occur (Additional file 7: Table S6), suggesting that their effect on our models would likely be small. Nonetheless, considering co-occurrence patterns could further refine the models.

Lastly, we explored one example of a unique aneuploidy pattern (chr13q) that is recurrently altered in opposite directions in different cancer types. In line with tumor suppressors and oncogenes being a major feature explaining aneuploidy patterns, we identified *KLF5* as a colorectal-specific dependency gene. Using an isogenic system of colorectal cancer cells with/without gain of chr13, we experimentally demonstrated that this aneuploidy is associated with increased expression and increased essentiality of *KLF5*. The finding that colorectal cells with trisomy 13 are more sensitive to *KLF5* depletion suggests positive selection for its gain, on top of a potential negative selection against a deleterious loss. We therefore propose that *KLF5* might explain why chr13q is commonly gained and rarely lost in colorectal cancer, unlike its recurrent loss across multiple other cancer types.

Overall, our study provides novel insights into the forces that shape the tissue-specific patterns of aneuploidy observed in human cancer and demonstrates the value of applying ML approaches to dissect this complicated question. Our results suggest that aneuploidy patterns are shaped by a combination of tissue-specific and non-tissue-specific factors. Negative selection in general and paralog compensation in particular play a major role in shaping the aneuploidy landscapes of human cancer and should therefore be computationally modeled and experimentally studied in the research of cancer aneuploidy.

## Methods

### Chromosome-arm aneuploidy patterns per cancer

Chromosome-arm events per cancer were defined according to GISTIC2.0 [24] for all (39) chromosome-arms in 24 cancer types for which data of the normal tissue of origin was available from GTEx [25]. GISTIC2.0 computed the probability of chromosome-arm events by comparing the observed frequency to the expected rate, while considering chromosome-arm length and other parameters [61]. A chromosome-arm was considered as gained or lost in a specific cancer if the *q*-value of its amplification or deletion, respectively, was lower than 0.05. Otherwise, the chromosome-arm was considered as neutral. In case the *q*-value of both amplification and deletion was lower than 0.05, decision was made based on the lower *q*-value. In case of a tie, the more frequent event was selected. GISTIC2.0 data, including *q*-values and frequencies, were downloaded from ref. [62]. Lastly, we analyzed co-incidence probabilities of chromosome-arm events per cancer. Co-incidence probabilities for chromosome-arms and cancers in our dataset were obtained from [37].The median fraction of chromosome-arm pairs with significant co-incidence per cancer was 2.05% (Additional file 7: Table S6). Hence, the impact of co-incidence on the models is expected to be small.

We also carried separate analyses of gain and loss of whole-chromosomes. A whole-chromosome was considered as gained if the *q*-value of the amplification of its two arms was lower than 0.05. Likewise, a whole-chromosome was considered as lost if the *q*-value of the deletion of its two arms was lower than 0.05.

### Construction of a features dataset of instances of chromosome-arm and cancer type pairs

For each chromosome-arm and cancer, we created features that were inferred from data of chromosome-arms, genes, cancer tissues and CCLs, and normal tissues (Fig. 1B, Additional file 2: Table S1). A schematic pipeline of the dataset construction appears in Additional file 1: Fig. S1. The different types of features are described below.

#### Features of chromosome-arms

Each chromosome-arm was associated with three types of features, including oncogene density, tumor suppressor gene density, and essential gene density. Oncogene density and tumor suppressor gene density per chromosome-arm were obtained from Davoli et al. [6]. Data of essential genes was obtained from Nichols et al. [26], where a gene was considered essential if its essentiality probability was > 0.8. The density of essential genes per chromosome-arm was calculated as the fraction of essential genes out of the protein-coding genes on that chromosome-arm. Next, we associated each instance of chromosome-arm and cancer type with features of that chromosome-arm.

#### Features of cancer tissues

Each instance of chromosome-arm and cancer type was associated with four types of cancer-related features, including transcriptomics, essentiality by CRISPR or RNAi in CCLs, and cancer-specific density of essential genes. Transcriptomics was based on transcriptomic profiles of 33 cancer types from TCGA [63] that were obtained from GDC Xena Hub v18.0 (updated 2019–08-28). Per cancer, we associated each gene with its median expression level in samples of that cancer. To avoid expression bias due to chromosome-arm gain or loss, the median expression of each gene was computed from samples where the chromosome-arm harboring the gene was neutral according to Taylor et al. [5]. Essentiality by CRISPR was based on CRISPR screens of 24 CCLs from the DepMap portal version 21Q1. Essentiality by RNAi was based on RNAi data of 20 CCLs from DepMap [27]. In each of these datasets, the score of each gene indicated the change, relative to control, in the growth rate of the cell line upon gene inactivation via CRISPR or RNAi. Accordingly, genes with negative scores were essential for the growth of the respective cell line. We associated each gene with its median essentiality score based on either CRISPR or RNAi per cell line. To reflect gene essentiality more intuitively, we reversed the direction of the scores (multiplied them by − 1), so that more essential genes had higher scores. To avoid bias due to chromosome-arm gain or loss, the median essentially of each gene was computed from samples where the chromosome-arm harboring the gene was neutral [5]. Cancer-specific density of essential genes was calculated as the fraction of essential genes (CRISPR-based essentiality score > 0.5) in a given CCL out of the protein-coding genes residing on that chromosome-arm.

#### Features of normal tissues

Each instance of chromosome-arm and cancer type was associated with 13 types of features that were derived from [55]. We associated each cancer type with the normal tissue in which it originates (Additional file 2: Table S1).

### Transcriptomics

Data of normal tissues included transcriptomic profiles of 54 adult human tissues measured via RNA-sequencing from GTEx v8 [25]. Each gene was associated with its median expression in each adult human tissue. Genes with median TPM > 1 in a tissue were considered as expressed in that tissue.

### Tissue-specific genes

Per gene, we measured its expression in a given tissue relative to other tissues using *z*-score calculation. Genes with *z*-score > 2 were considered tissue-specific. Lastly, we associated each chromosome-arm and tissue with the density of tissue-specific genes.

### PPI features

Each gene was associated with the set of its PPI partners. We included only partners with experimentally detected interactions that were obtained from MyProteinNet web-tool [64]. Per each tissue, we associated each gene with four PPI-related features:

(i) “Number PPIs” was set to the number of PPI partners that were expressed in that tissue.
(ii) “Number elevated PPIs” relied on preferential expression scores computed according to [28] and was set to the number of PPI partners that were preferentially expressed in that tissue (preferential expression > 2, [65].
(iii) “Number tissue-specific PPIs” was set to the number of PPI partners that were expressed in that tissue and in at most 20% of the tissues.
(iv) “Differential PPIs” relied on differential PPI scores per tissue from The DifferentialNet Database [28] and was set to gene’s median differential PPI score per tissue. If the gene was not expressed in a given tissue, its feature values in that tissue were set to 0.

### Differential process activity features

Differential process activity scores per gene and tissue were obtained from [30]. The score of a gene in a given tissue was set to the median differential activity of the Gene Ontology (GO) processes involving that gene. The differential activity was relative to the activity of the same processes in other tissues.

### eQTL features

eQTLs per gene and tissue were obtained from GTEx [25]. Each gene was associated with the *p*-value its eGene in that tissue.

### Paralog compensation features

Each gene was associated with its best matching paralog according to Ensembl-BioMart. Per tissue, the gene score was set to the median expression ratio of the gene and its paralog, as described in [31, 32]. Accordingly, high values mark genes with low paralog compensation.

### Development features

Transcriptomic data of seven human organs measured at several time points during development were obtained from [66]. We united time points into time periods including fetal (4–20 weeks post-conception), childhood (newborn, infant, and toddler), and young (school, teenager and young adult). Per organ, we associated each gene with its median expression level per period. Next, we created an additional feature that reflected the expression variability of each gene across periods.

#### Transforming gene features into chromosome-arm features

Some of the features described above referred to genes. To create chromosome-arm-based features, we grouped together genes that were located on the same chromosome-arm [67]. Next, to highlight differences between tissues, for each feature, we associated a gene with its value in that tissue relative to other tissues. Features that were already tissue-relative, including “Differential PPIs” and “Differential process activity,” were maintained. Other features were converted into tissue-relative values via a *z*-score calculation (see Eq. 1). Lastly, per feature, we ranked genes by their tissue-relative score and associated each chromosome-arm with the median score of the genes ranking at the top 10% (Additional file 1: Fig. S2). Transcriptomic features in the testis and whole blood were highly distinct from other tissues; we normalized all transcriptomic features per tissue. To reflect paralog compensation more intuitively, we reversed the direction of the resulting features (multiplied them by − 1), so that genes with higher compensation had higher scores.

1 $${\forall t\in T,\forall g\in G: z\mathrm{ score}}_{g}^{t}= \frac{[{v}_{g}^{t}-{\text{mean}}({v}_{g}^{\forall {t}^{\mathrm{^{\prime}}}\in T})]}{\sigma [{v}_{g}^{\forall {t}^{\mathrm{^{\prime}}}\in T}]}$$

*T* denotes the set of tissues,* G* denotes the set of genes,* v* denotes the value of the feature, and σ denotes the standard deviation.

#### Construction of the final dataset

The features described above referred to chromosome-arms in cancers, CCLs, and normal tissues. To create chromosome-arm features per cancer, we associated each cancer with the chromosome-arm features of its tissue of origin and CCL (Additional file 2: Table S1). For features of normal tissues where multiple sub-regions were sampled (e.g., skin sun-exposed and not sun-exposed, or brain sub-regions), we set the chromosome-arm values to their median across sub-regions. The final dataset contained features for all 936 instances of 39 chromosome-arms and 24 cancers for which the cancer’s normal tissue of origin was available in GTEx [25] (Additional file 2: Table S1). We assessed the similarity between every pair of features using Spearman correlation (Additional file 1: Fig. S3A). We assessed whether chromosome-arm and cancer type instances had similar feature values using PCA (Additional file 1: Fig. S3C).

### ML application to model chromosome-arm and cancer aneuploidy

Below we describe the ML method used for aneuploidy classification and the SHAP (SHapley Additive exPlanations) analysis of feature importance that was used to interpret the resulting models.

#### Aneuploidy ML classification models

We constructed two ML models: a gain model that compared between gained and unchanged (neutral) chromosome-arms and a loss model that compared between lost and unchanged (neutral) chromosome-arm.

#### ML comparison and implementation

Per model, we tested several ML methods, including logistic regression, XGBoost, gradient boosting, random forest, and bagging. All ML methods were implemented using the Scikit-learn python package [68], except for XGB, which was implemented using the Scikit-learn API of the XGBoost package [69]. To assess the performance of each model, we used tenfold cross-validation. Then, we calculated the au-ROC and the au-PRC. Each point on the curve corresponded to a particular cutoff that represented a trade-off between sensitivity and specificity and between precision and recall, respectively.

#### SHAP analysis of feature importance

To measure the contribution and importance of the different features, we used SHAP algorithm [70]. SHAP is a game-theoretic approach to explain the output of ML models: for each feature, SHAP assigns a contribution value to each instance of chromosome-arm and cancer type. It then estimates the contribution of that feature to the model by the average absolute SHAP values of all instances. Per model, we created the SHAP plots corresponding to feature contribution and directionality. In both, features were ordered by their importance to the model (top meaning most contributing). We also visualized the directionality of each feature using arrows in the SHAP bar plot. The direction of the arrow showed whether the highest values of that feature (top 50%) corresponded to a chromosome-arm event (gain or loss, right) or to neutrality (left).

#### Robustness analyses

We analyzed the robustness of the models and their interpretation with respect to internal parameters used to generate the features and the hyperparameters of the ML models. For feature generation, we used top 10% of genes with highest values to calculate each gene-based chromosome-arm feature. We therefore reconstructed features by also using the top 1%, 5%, 15%, and 20% of the genes. We then assessed the performance of each method using tenfold cross-validation. In all cases, method performance was similar (Additional file 3: Table S2). SHAP analysis of the best performing method per case showed similar results with respect to the topmost contributing features and their directionality (Additional file 1: Fig. S7). For robustness to parameter choices, we tuned the hyperparameters per ML method separately for the gain model and for the loss model, and repeated model construction and interpretation. Tuning was optimized for precision and performed using the “RandomizedSearch” function of sklearn python package, with number of sampled parameters (iterations, n_iter) set to 200 and tenfold cross-validation. Best parameters per method and model and their performance appear in Additional file 1: Fig. S8A,B. Performance was only slightly improved, and interpretation of the best performing models revealed similar results (Additional file 1: Fig. S8C).

Lastly, we tested if the most important features per model were driven by a small subset of chromosome-arm and cancer type instances. For that, per model, we focused on the five most important features and identified instances with the top contributions to these features. An instance was considered a top contributor if its SHAP value for that feature that was among the 10% positive SHAP values (i.e., was a potential driver of the gain or loss) or the 10% negative SHAP values (i.e., was a potential driver of neutrality). The SHAP value for each instance and feature appears in Additional file 4: Table S3. The list of instances and the features that they contributed to appears in Additional file 5: Table S4. We then associated each instance with the number of features in which it was a top contributor. Next, we tested the impact of the strongest potential driver instances on the five most important features of the model. This was done by excluding from the dataset chromosome-arm and cancer type instances that were top contributors to at least three of the five features and repeating the construction and interpretation of each model using the revised dataset.

### Correlation analysis

We correlated between feature values and the frequency of chromosome-arm gain or loss. The frequency of chromosome-arm gain/loss in cancers was obtained from GISTIC2.0 [24]. The frequency of chromosome-arm gain/loss in CCLs were obtained from [37]. Per chromosome-arm, its gain (loss) frequency was set to the median gain (loss) across cancers or CCLs. The feature value was set to median across cancers or CCLs. We used Spearman correlation, and *p*-values were adjusted using Benjamini–Hochberg procedure [71].

### Paralog compensation analysis

For each cancer type and chromosome-arm, we considered all paralog pairs in which one of the genes resides on that chromosome-arm. We focused on recurrently lost genes per cancer type as defined by GISTIC2.0 [24]. We divided those genes by their minimal CRISPR essentiality score in CCLs that match the same cancer type (Additional file 2: Table S1). Genes with a score ≤ − 0.5 were considered essential, and genes with a score ≥ − 0.3 were considered non-essential. Other genes were considered intermediate. Per gene, we checked whether its paralog was recurrently gained, lost, or neutral, in the same cancer, as detailed in Additional file 1: Fig. S17A.

### Chromosome-arm aneuploidy patterns in CCLs

Aneuploidy patterns were available for all (39) chromosome-arms in 14 CCLs from [37]. A chromosome-arm was considered as gained or lost in a CCL if the *q*-value of its amplification or deletion, respectively, was smaller than 0.15 (in case of ties, decision was made based on the lower *q*-value). In case of equal significant *q*-values, a chromosome-arm was considered as gained or lost based on their frequencies. Otherwise, the chromosome-arm was considered as neutral.

#### Construction of a feature dataset of instances of chromosome-arm and CCL pairs

The features dataset was similar to the dataset created for cancers, with the following exceptions. In features of cancer tissues, we replaced the transcriptomic features of cancers with transcriptomic features of CCLs. We obtained transcriptomic data of 25 CCLs from DepMap [27] and constructed the feature values per chromosome-arm and CCL as described above per chromosome-arm and cancer. Development features were removed since only a small number of CCLs had a matching organ. The final dataset contained features for all instances of 39 chromosome-arms and 10 CCLs for which the cancer’s normal tissue of origin was available in GTEx.

### Cell culture

DLD1-WT cells and DLD1-Ts13 cells were cultured in RPMI-1640 (Life Technologies) with 10% fetal bovine serum (Sigma-Aldrich) and 1% penicillin–streptomycin-glutamine (Life Technologies). Cells were incubated at 37 °C with 5% CO2 and passaged twice a week using Trypsin–EDTA (0.25%) (Life Technologies). Cells were tested for mycoplasma contamination using the MycoAlert Mycoplasma Detection Kit (Lonza), according to the manufacturer’s instructions.

### qRT-PCR

Cells were harvested using Bio-TRI® (Bio-Lab) and RNA was extracted following manufacturer’s protocol. cDNA was amplified using GoScript™ Reverse Transcription System (Promega) following manufacturer’s protocol. qRT-PCR was performed using Sybr® green, and quantification was performed using the ΔCT method. The following primer sequences were used: human *KLF5*, forward, 5' ACACCAGACCGCAGCTCCA 3' and reverse 5' TCCATTGCTGCTGTCTGATTTGTAG 3', human *NEK3*, forward, 5’ TACCCAAATGTGCCTTGGAG 3’, reverse 5’ ATCGGATTGGAGAGAAGACG 3’, human *TTC7A*, forward 5’ CTCGTGACCTGCAGACAAG 3’, reverse 5’ GGCTCCTAAAGTCTCCCAGC 3’.

### siRNA transfection

For siRNA experiments, cells were plated in 96-well plates at 6000 cells per well and treated with compounds 24 h later. The cells were transfected with 15 nM siRNA against *KLF5* (ONTARGETplus SMART-POOL®, Dharmacon) or with a control siRNA at the respective concentration (ONTARGETplus SMART-POOL®, Dharmacon) using Lipofectamine® RNAiMAX (Invitrogen) following the manufacturer’s protocol. Alternatively, for siRNA experiments against *NEK3* and *TTC7A*, and for additional *KLF5* experiments, cells were plated in 6-well plates at 400,000 cells per well and treated with compounds 24 h later. The cells were transfected with 30 nM against *NEK3* and *TTC7A* or with 5 nM and 10 nM against *KLF5*; 48 h post seeding, the cells were split and plated in 96-wells at 10,000 cells per well. The effect of the knockdown against *KLF5*, *NEK3*, or *TTC7A* on cell viability/proliferation was measured by live cell imaging using Incucyte® (Satorius) or by the MTT assay (Sigma M2128) at 72 h (or at the indicated time point) post-transfection; 500 µg/mL MTT salt was diluted in complete medium and incubated at 37°C for 2 h. Formazan crystals were extracted using 10% Triton X-100 and 0.1 N HCl in isopropanol, and color absorption was quantified at 570 nm and 630 nm (Alliance Q9, Uvitec).

### Cancer cell line and tumor data analysis

mRNA gene expression values, arm-level CNAs, CRISPR, and RNAi dependency scores (Chronos and DEMETER2 scores, respectively) were obtained from DepMap 22Q4 release (www.depmap.org). Effect size, *p*-values, and *q*-values (Fig. 4A,C,E, Fig. 5C) were taken directly from DepMap and were calculated as described in Tsherniak et al. TCGA mRNA gene expression values were obtained using the Xena browser [63]. Tumor arm-level alterations were retrieved from Taylor et al. 2018, *Cancer Cell.* Effect size, Spearman’s R and *p*-values in Fig. 4G and Fig. 5F were calculated using R functions. All colorectal cancer cell lines (*n* = 85) and colorectal tumors (*n* = 434) were included in the analyses.

The analyses that led to our choice of the paralog pair *UCHL3*-*UCHL1* are summarized in Additional file 6: Table S5. In the left column are the paralogs that reside on chr-13q, which is frequently gained; in the adjacent column are the respective paralogs that reside on commonly lost chromosomes. The following columns describe the Spearman correlation between each paralog pair and the respective *p*-value. The right-hand columns describe the effect size of chr-13q paralogs’ gene expression between CRC cell lines with and without chr13q gain. Our criteria for finding appropriate paralog pairs for further analysis were as follows: firstly, to have a high expression of the chr-13q paralogs in CRC cell lines. Secondly, we aimed to reach a significant correlation between chr13q-residing genes and the essentiality of their paralogs.

### Statistical analyses

Statistical analysis was performed using GraphPad PRISM® 9.1 software. Details of the statistical tests were reported in figure legends. Error bars represent SD. All experiments were performed in at least three biological replicates.

### Supplementary Information

**Additional file 1: Supplementary Figures.** This file contains Supplementary Figures S1-S17.

**Additional file 2: Table S1.** Association of TCGA cancer types with normal tissues-of-origin and matching cell lines.

**Additional file 3: Table S2.** The auROC and auPRC performance of ML models whose features were calculated using distinct percentages of genes.

**Additional file 4: Table S3.** SHAP value per feature of each instance of chromosome-arm and tumor type in the gain and loss models.

**Additional file 5: Table S4.** Potential driver instances of each feature in the gain and loss models, and their frequencies.

**Additional file 6: Table S5.** Correlations between chr-13q residing genes and the essentiality of their paralogs.

**Additional file 7: Table S6.** Co-incidence of arm-level events in the different cancer types, and their frequencies.

**Additional file 8.** Review history

## Data Availability

The code for all the analyses is available on GitHub [72]. The datasets that were processed to build the dataset for the ML methods are available on Zenodo [73]. This includes features of normal tissues that were extracted from TRACE [74], TCGA expression data of the different cancer types that were obtained from Xena [75], and CRISPR and RNAi datasets that were obtained from DepMap [76].
