# Supplementary material for: Machine-learning analysis reveals an important role for negative selection in shaping cancer aneuploidy landscapes
Source: Genome Biol. 2024 Apr 15;25:95. doi: 10.1186/s13059-024-03225-7 (PMC11020441; doi:10.1186/s13059-024-03225-7)
Supplement: Supplementary file 1 — Additional file 1: Supplementary Figures. This file contains Supplementary Figures S1-S17. [file 13059_2024_3225_MOESM1_ESM.pdf]

Additional file 1: Supplementary figures

This file contains Figures S1-S17.

Machine-learning analysis of factors that shape cancer aneuploidy landscapes reveals an important role for negative selection

Juman Jubran[1,*], Rachel Slutsky[2,*], Nir Rozenblum[2], Lior Rokach[3], Uri Ben-David[2,#], Esti Yeger-Lotem[1,4,#]

Affiliation:

[1] Department of Clinical Biochemistry and Pharmacology, Ben-Gurion University of the Negev, Beer Sheva 84105, Israel.

[2] Department of Human Molecular Genetics and Biochemistry, Faculty of Medicine, Tel Aviv University, Tel Aviv, Israel

[3] Department of Software & Information Systems Engineering, Ben-Gurion University of the Negev, Beer Sheva 84105, Israel.

[4] The National Institute for Biotechnology in the Negev, Ben-Gurion University of the Negev, Beer Sheva 84105, Israel.

* Equally-contributing first authors.

# Equally-contributing last authors.
Correspondence should be addressed to: ubendavid@tauex.tau.ac.il or estiyl@bgu.ac.il

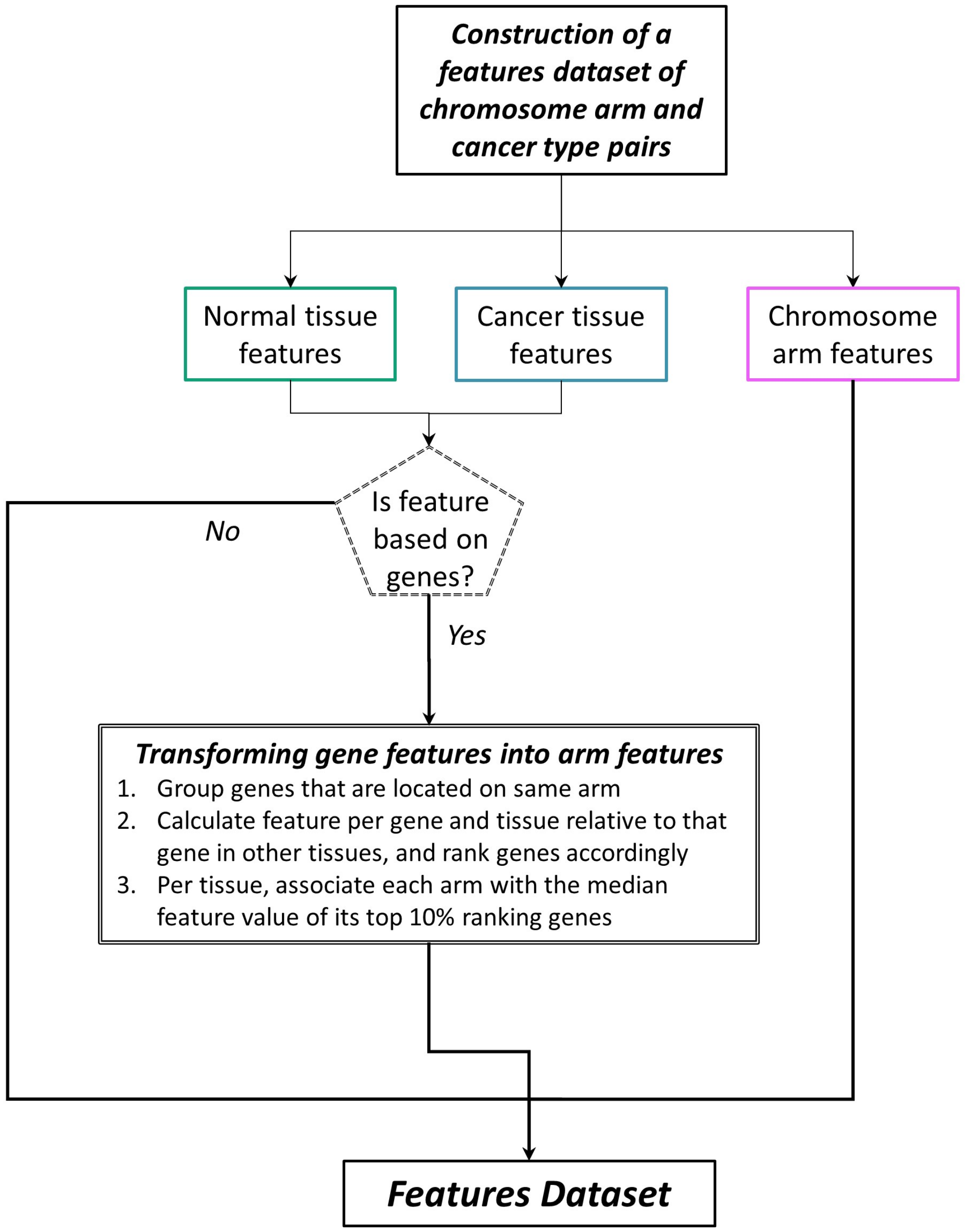

Fig. S1. Workflow for constructing the features dataset.
We associated each instance of a chromosome-arm and cancer type pair with three categories of features: chromosome-arm, normal tissues and cancer tissues features. Features belonging to the chromosome-arm category were independent of cancer types. Features belonging to the categories of normal and cancer tissue features were either inferred from data of the entire chromosome-arm, in which case no transformation was needed, or were inferred from data of individual genes, in which case these data were collated per chromosome-arm and transformed into chromosome-arm-based features.

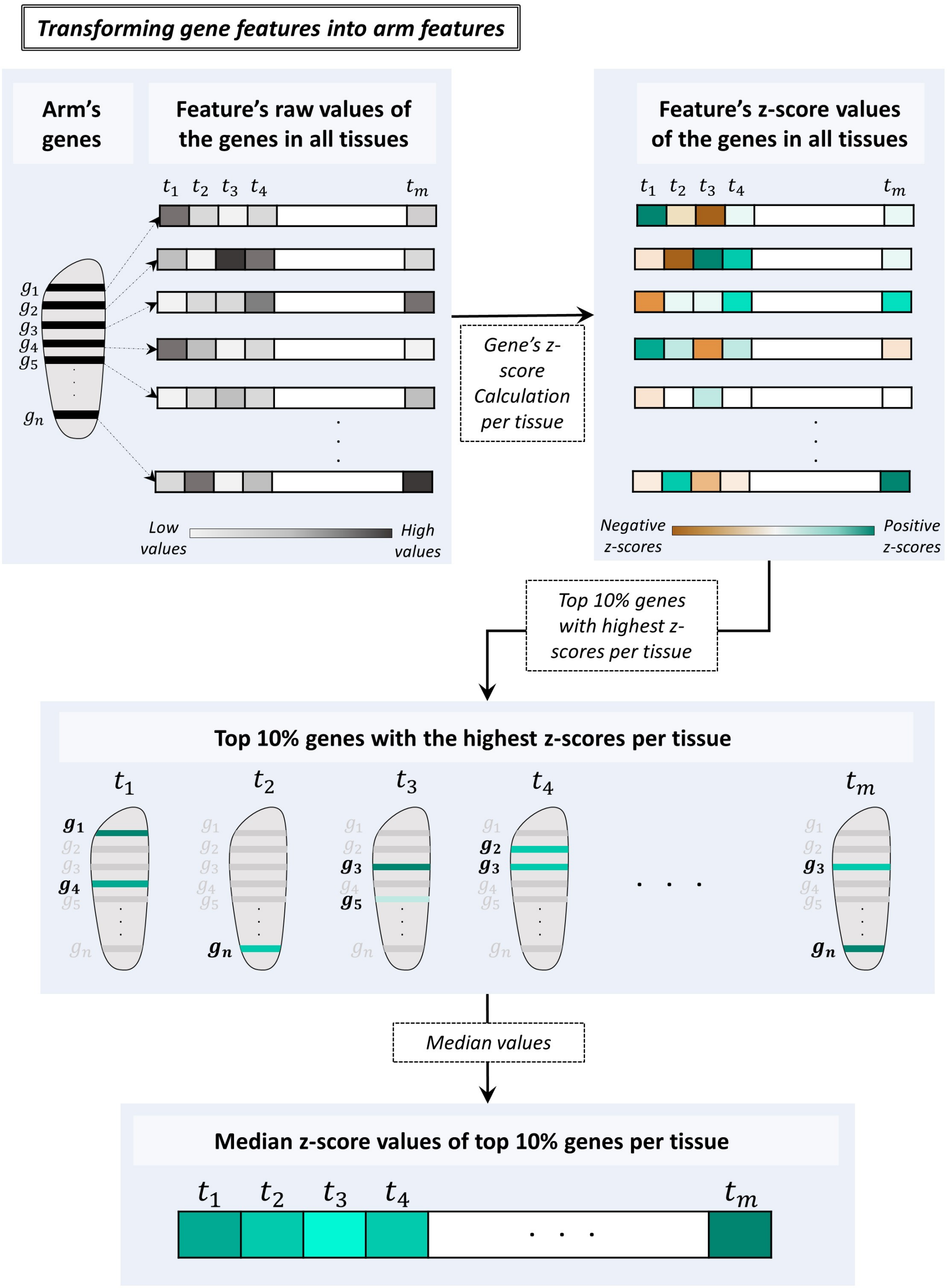

Fig. S2. Workflow for transforming gene-related features into chromosome-arm related features. For each gene-related feature and chromosome-arm, genes located on that chromosome-arm were grouped. Their raw values in the different tissues were transformed into z-scores per gene and tissue. Then, each tissue was associated with the median of the top 10% genes with the highest z-score.

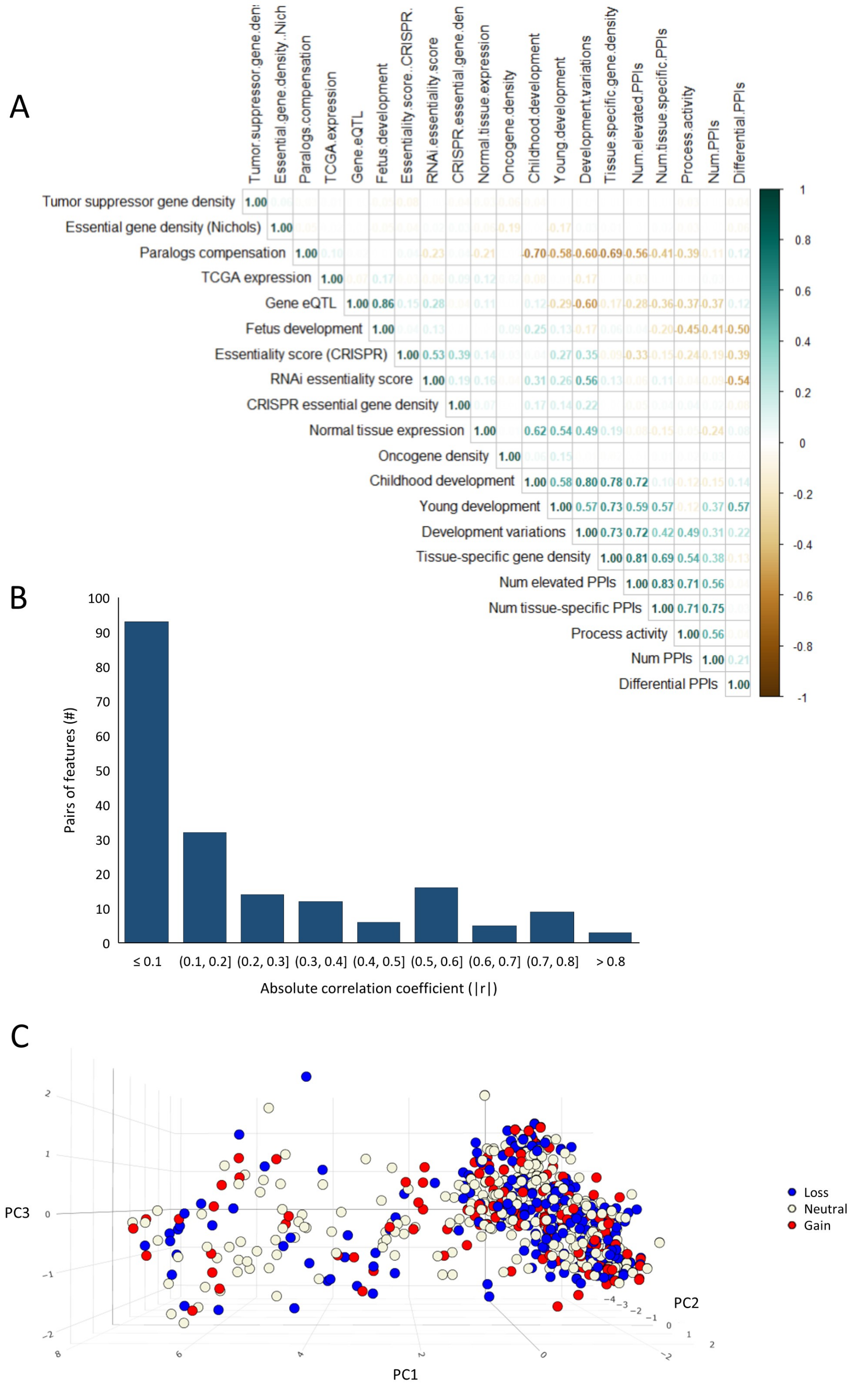

Fig. S3. Unravelling patterns and relationships across features.

A. Assessment of the similarity between every pair of features using Spearman correlation. Positive correlations are presented in green and negative correlations are presented in gold.

B. The distribution of all (190) pairs of features by their absolute correlation values. Most pairs had low absolute correlation values.

C. Principal component analysis (PCA) of all instances of chromosome-arms and cancer type pairs, according to their feature values. Instances did not cluster by their aneuploidy pattern.

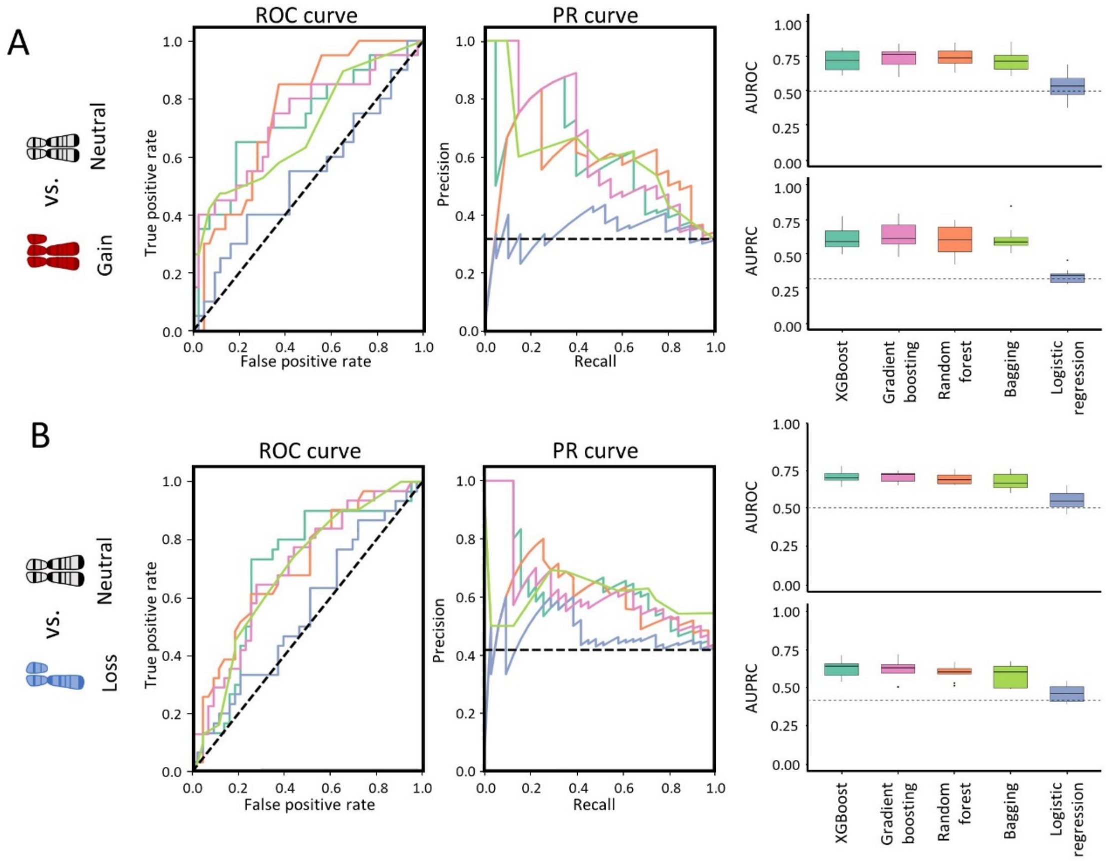

Fig. S4. Performance of different ML methods for predicting aneuploidy in cancer.

Performance in 10-fold cross-validation of each method was measured by calculating auROC and auPRC. Curves represent the median auROC and auPRC of each method. Boxplots represent the results of auROC and auPRC of all 10 folds.

A. Classification between a gain of an arm versus the neutral arms. Gradient boosting method performed best in terms of the auROC and auPRC, and was henceforth used to model chromosome-arm gains.

B. Models were applied to classify loss of chromosome-arms versus neutral chromosome-arms. XGBoost performed best in terms of the auROC and auPRC, and was henceforth used to model chromosome-arm loss.

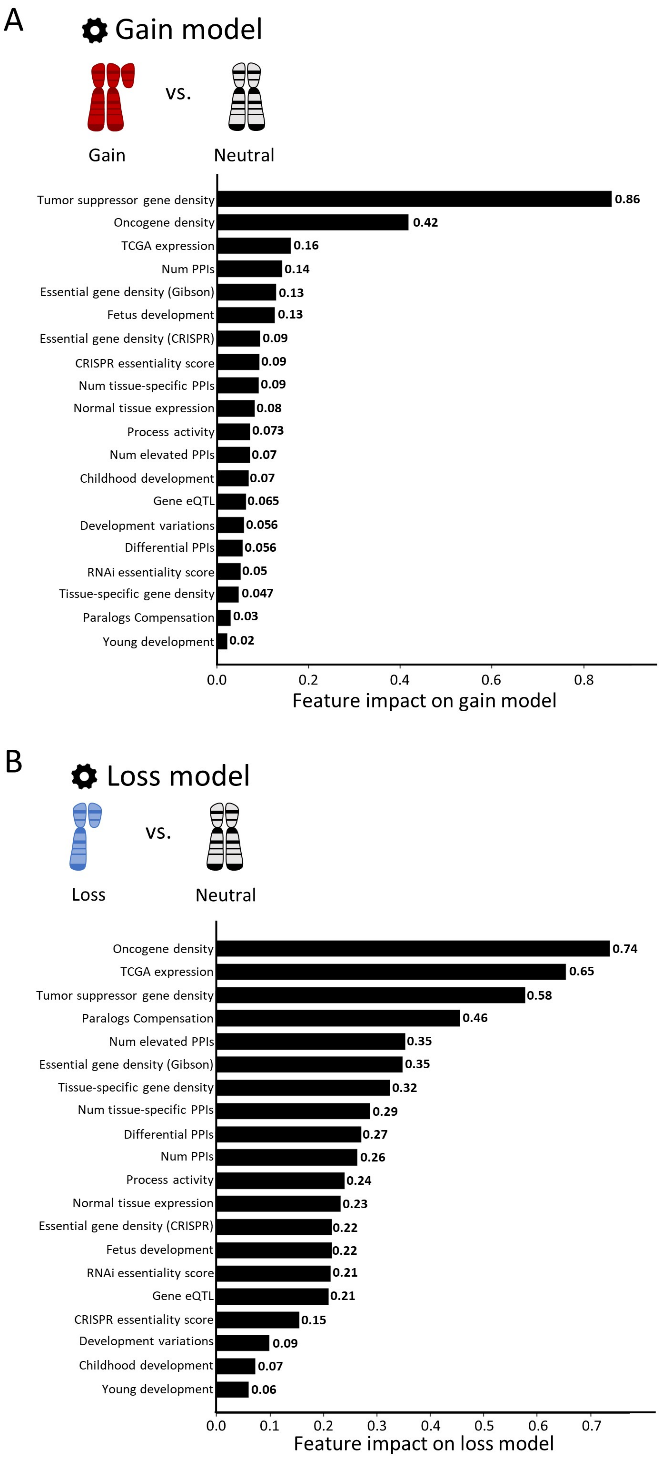

Fig. S5. Contribution of all the features to gain and loss models in cancer.
Features are ordered from bottom to top by their increased average absolute contribution to the model. A. Gain model. B. Loss model.

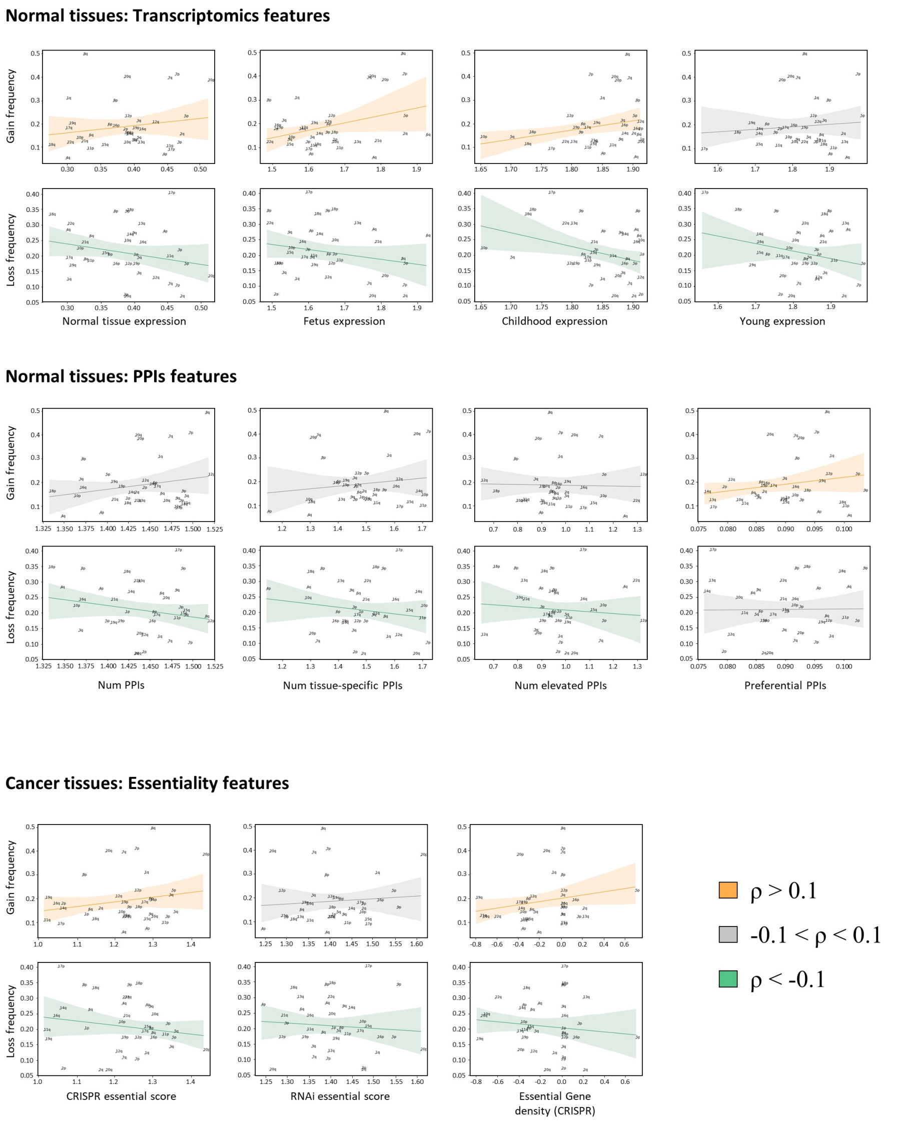

Fig. S6. Correlation between different features and the frequencies of chromosome-arms gain and loss.

The confidence interval was colored by the Spearman correlation value. Positive correlations (r>0.1) appear in orange, negative correlations (r<-0.1) appear in green, otherwise they appear in grey.

A. Transcriptomics features measured in adult [1], fetal [2], child [2] and young subjects [2] were modestly positively correlated with chromosome-arm gain frequency and modestly negatively correlated with chromosome-arm loss. Adult tissues: ρ=0.12, ρ=-0.22 respectively. Fetal tissues: ρ=0.15, ρ=-0.17 respectively. Child tissues: ρ=0.2, ρ=-0.28 respectively. Young tissues: ρ=-0.03, ρ=-0.16 respectively.

B. PPI features were generally uncorrelated with chromosome-arm gain frequency and modestly negatively correlated with chromosome-arm loss frequency. Num PPIs: ρ≈0, ρ=-0.18 respectively. Num tissue-specific PPIs: ρ≈0, ρ=-0.13 respectively. Num elevated PPIs: ρ≈0, ρ=-0.13 respectively. Differential PPIs: ρ=0.18, no correlation respectively.

C. Gene essentiality features were modestly positively correlated with chromosome-arm gain frequency and modestly negatively correlated with chromosome-arm loss frequency. CRISPR essential score: ρ=0.13, ρ=-0.14 respectively. RNAi essential score: ρ≈0, ρ=-0.12 respectively. Essential gene density (CRISPR): ρ=0.18, ρ=-0.1 respectively.

A

Gain vs. Neutral	
Model	Best parameters
XGBoost	eta=0.01, min_child_weight=5, gamma=2.5, subsample=1, colsample_bytree=0.6, max_depth=6, alpha=1
Gradient Boosting	learning_rate=0.05, n_estimator=100, subsample=0.8, min_samples_split=2, min_sample_leaf=10
Random Forest	bootsrtrap=True, max_depth=30, min_sample_leaf=2, min_samples_split=10, n_estimator=400
Bagging	n_estimator=80, max_samples=0.3, max_features=0.8, bootstrap=True
Logistic regression	penalty=l1, C=3

Loss vs. Neutral	
Model	Best parameters
XGBoost	eta=0.1, min_child_weight=1, gamma=1, subsample=1, colsample_bytree=0.6, max_depth=5, alpha=1
Gradient Boosting	learning_rate=0.05, n_estimator=300, subsample=0.8, min_samples_split=2, min_sample_leaf=5
Random Forest	bootsrtrap=True, max_depth=None min_sample_leaf=3, min_samples_split=10, n_estimator=200
Bagging	n_estimator=50, max_samples=0.3, max_features=1, bootstrap=False
Logistic regression	penalty=l1, C=3

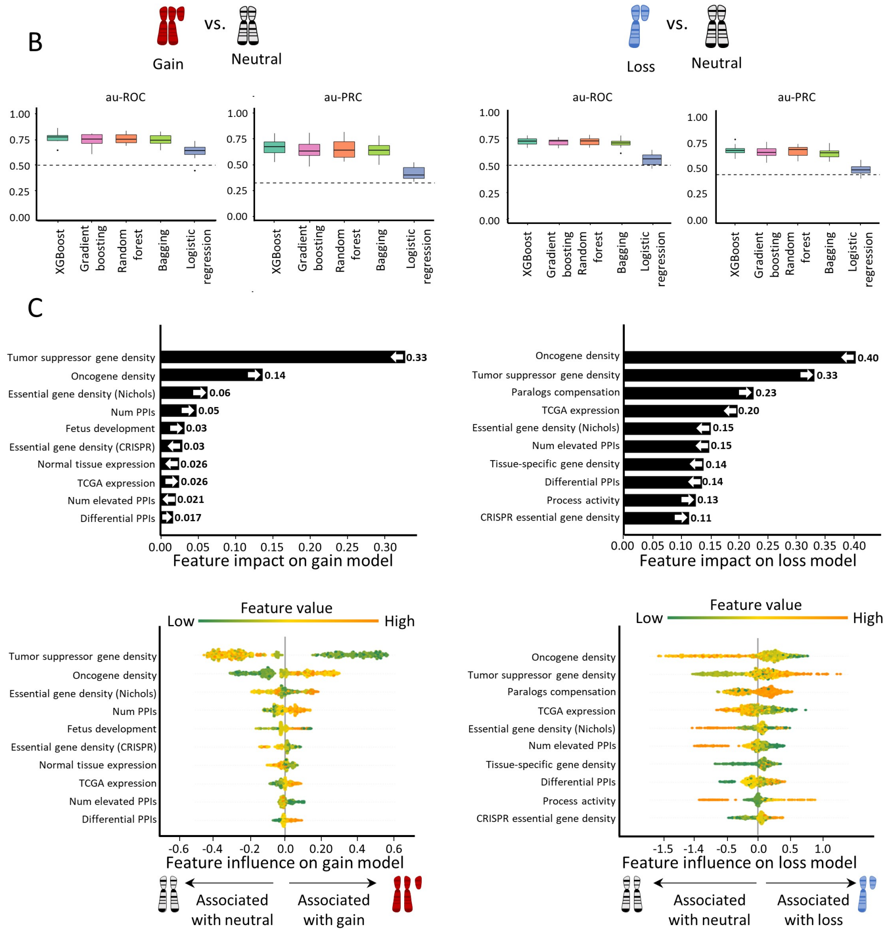

Fig. S7. Robustness of the topmost contributing features to the percentage of genes that were used to set feature values.

Gain and loss models were recalculated and interpreted after modifying the percentage of genes that were used to set feature values from 10% (original) to 1%, 5%, 15%, and 20%. Similar to the original gain model, all gain models shared 'TSG density' and 'OG density' as the first and second most contributing features, respectively. 'Essential gene density' was the top fourth and 'TCGA expression' was among the top five most contributing features, except for the model based on 5% of the genes. Similar to the original loss model, all loss models shared 'OG density' and 'TSG density' as the first and second most contributing features, respectively. 'TCGA expression' and 'paralogs compensation' were among the top six and top ten most contributing features, respectively. Hence, the top contributing features were generally robust to the percentage of genes that were used to set feature values per model. Taken together, these results support the role of negative selection in determining aneuploidy patterns.

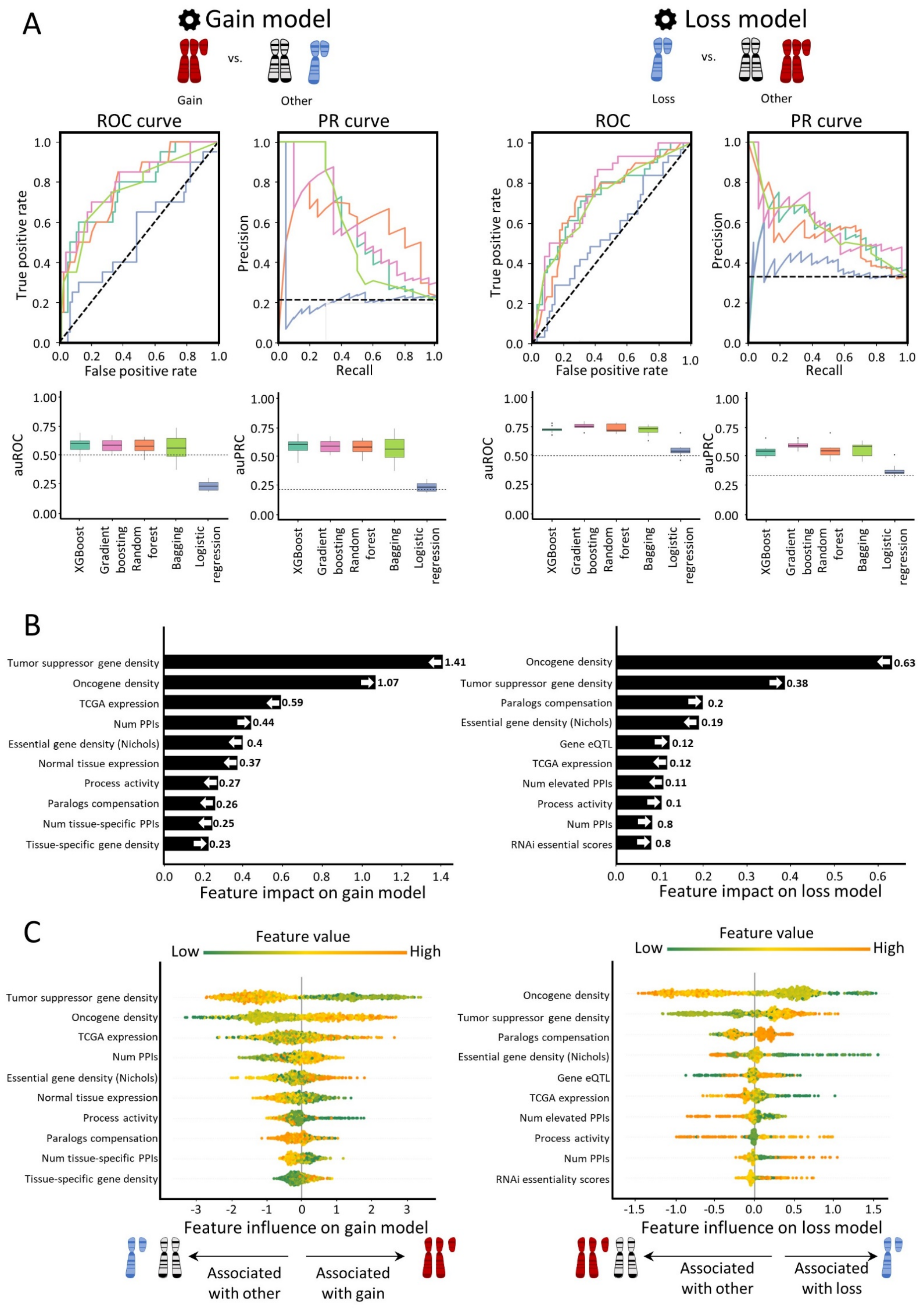

Fig. S8. Model robustness to hyperparameter tuning.

A. Parameters tuned for best precision per model and ML method.

B. Performance of each method upon using the optimized parameters from panel A. Performance was measured by calculating auROC and auPRC using 10-fold cross-validation. The best performing method was XBGoost, with auROC and auPRC that were slightly improved relative to untuned models (in parenthesis): Gain model: auROC 0.77 (0.74), auPRC 0.67 (0.63). Loss model: auROC 0.73 (0.7), auPRC 0.66 (0.63).

C. The contribution of the topmost contributing features to the gain model (left) and loss model (right). See Fig. 2A-B for a detailed description. In the gain model, 'TSG density' was the topmost contributing feature, with a contribution that was over 2-fold higher than the second most contributing feature, 'OG density', confirming the role of negative selection. The topmost contributing features of the loss model preserved the ranking and directionality of the original untuned model (Fig. 2).

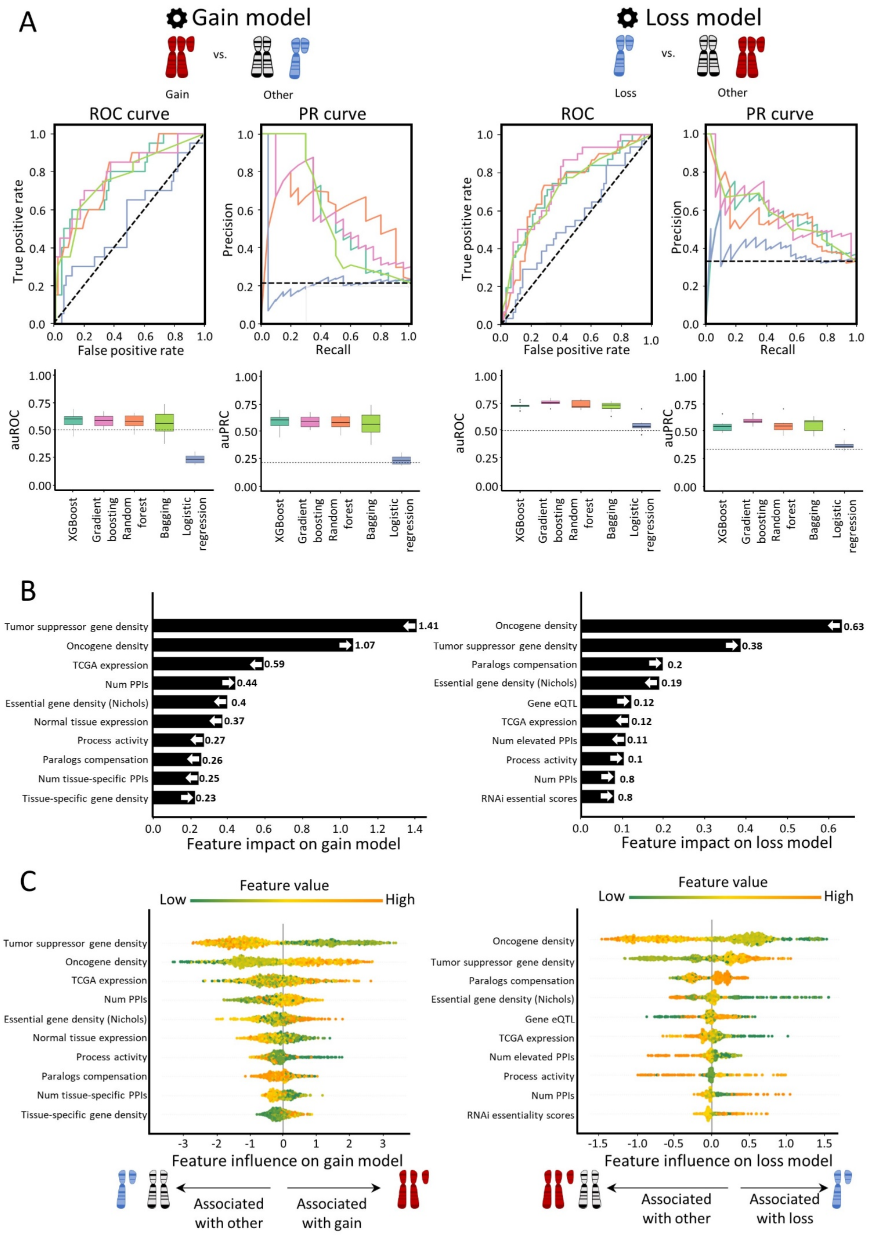

Fig. S9. ML analysis of chromosome-arm gain versus all other events and chromosome-arm loss versus all other events.

A. ML models were applied to classify chromosome-arm gain versus loss or neutrality (left), and chromosome-arm loss versus gain or neutrality (right). Performance was measured by calculating auROC and auPRC using 10-fold cross-validation. In the gain model, XGBoost achieved the best auROC (0.77) and auPRC (0.59), and was henceforth used to model chromosome-arm gain. In the loss model, gradient boosting achieved the best auROC (0.76) and auPRC (0.59), and was henceforth used to model chromosome-arm loss.

B. The average absolute contribution of each feature to the gain versus all other events model (left) and the loss versus all other events model (right). See Fig. 2A for a detailed description.

C. The contribution of the topmost contributing features to the gain versus all other events model (left) and to the loss versus all other events model (right). See Fig. 2B for a detailed description. The topmost contributing features of the gain and loss models generally preserved the ranking and directionality of the original models (Fig. 2).

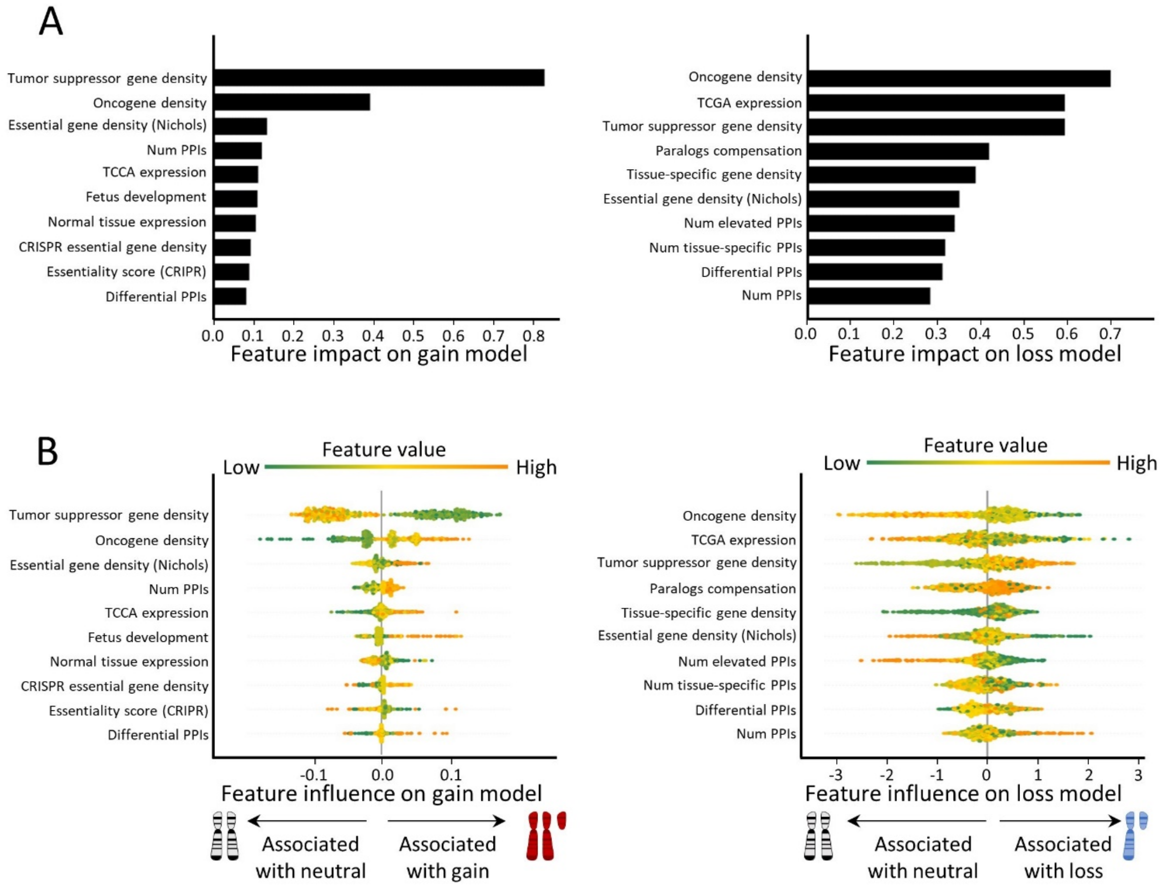

Fig. S10. Model robustness upon removing the top contributing chromosome-arm and cancer type instances.

The list of 27 and 14 instances removed from the construction of the gain and loss models, respectively, appears in Table S4.

A. The average absolute contribution of each feature to the gain model (left) and to the loss model (right). See Fig. 2A for a detailed description.

B. The contribution of the topmost contributing features to the gain model (left) and to the loss model (right). See Fig. 2B for a detailed description. The order and directionality of the topmost contributing features generally agreed with the original gain and loss models (Fig. 2).

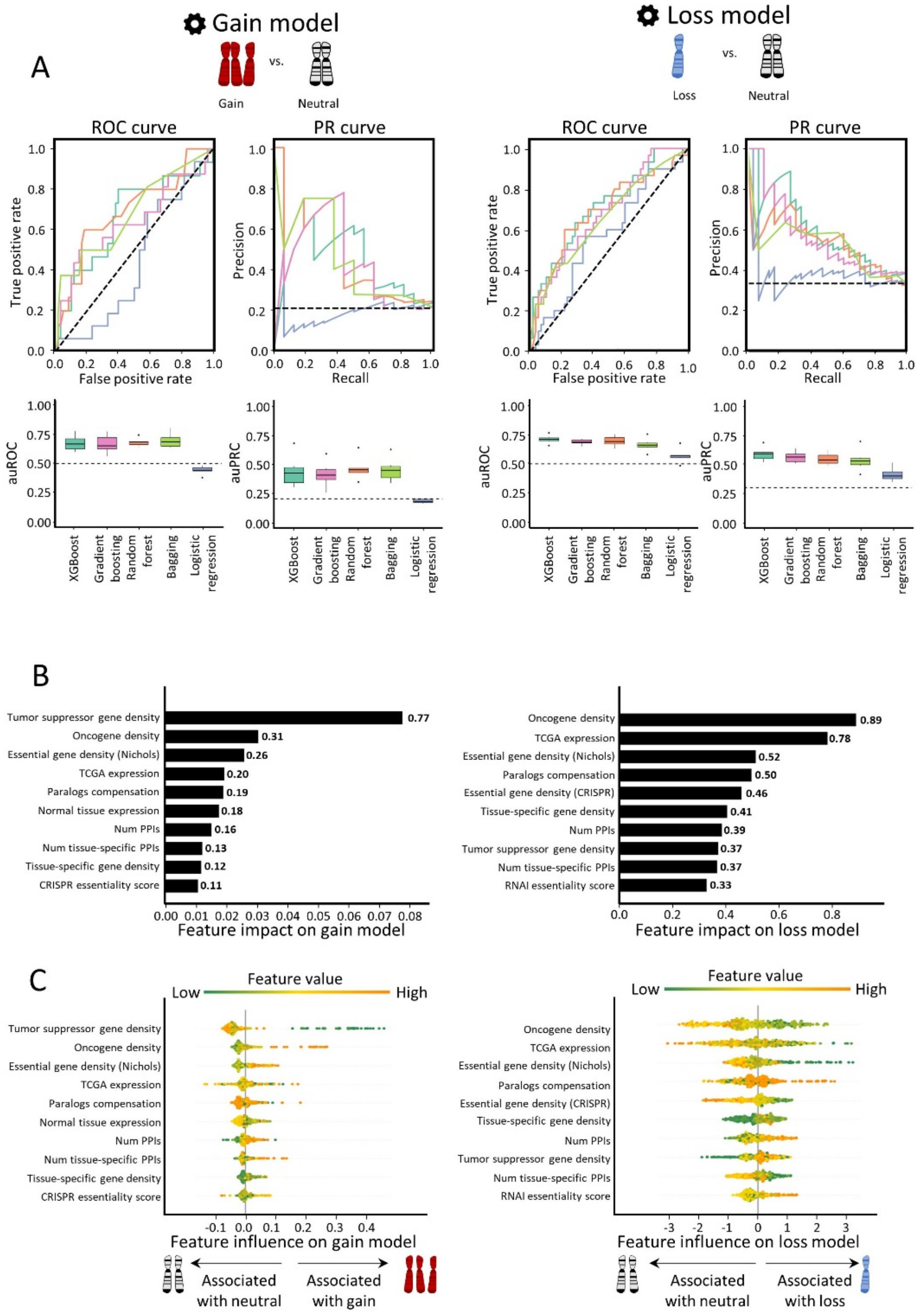

Fig. S11. ML analysis of whole-chromosome gain (monosomy) and whole-chromosome loss model.
A. Models were applied to classify whole-chromosome gain versus neutrality (trisomy, left), and whole-chromosome loss versus neutrality (monosomy, right). Performance was measured by calculating auROC and auPRC using 5-fold cross-validation. In the trisomy model, random forest achieved the best auROC (0.69) and auPRC (0.47, expected 0.21). In the monosomy model, XGBoost achieved the best auROC (0.71) and auPRC (0.59, expected 0.34).
B. The average absolute contribution of each feature to the trisomy model (left) and monosomy model (right).
C. The contribution of the topmost contributing features to the trisomy model (left) and the monosomy model (right). See Fig. 2B for a detailed description. The order and directionality of the features generally agree with model of primary tumors (Fig. 2).

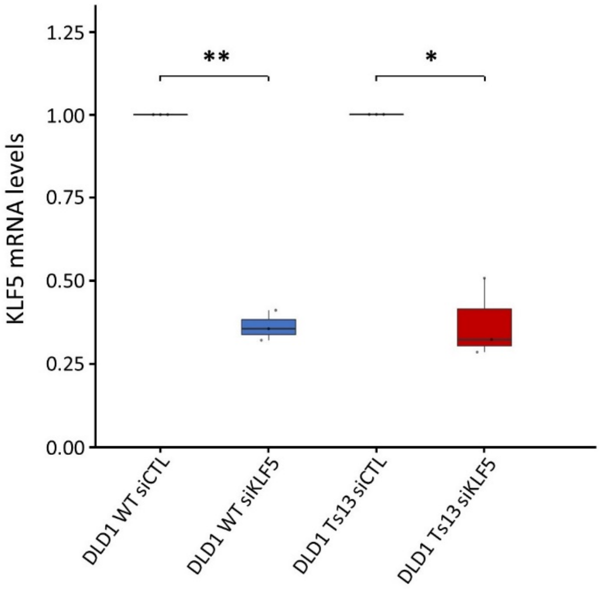

Fig. S12: Validation of *KLF5* knockdown in DLD1 isogenic cell lines

Comparison of *KLF5* mRNA levels between DLD1-WT and DLD1-Ts13 after siRNA treatment against *KLF5*. Both DLD1-WT and DLD1-Ts13 reached efficient knockdown. n=3 independent experiments. **, p=0.005 and *, p=0.003; One-sample t-test.

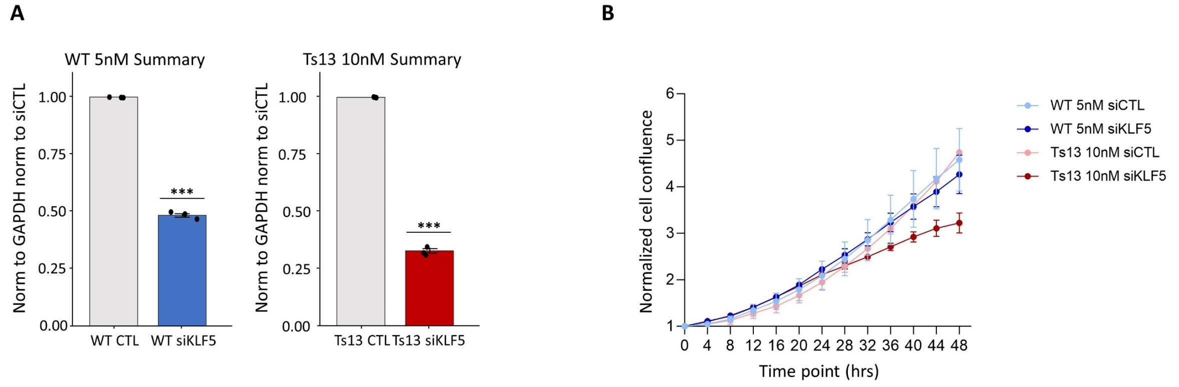

Fig. S13: **Validation of absolute *KLF5* knockdown in DLD1 isogenic cell lines.**

A. Comparison of the absolute amount in *KLF5* mRNA levels between DLD1-WT and DLD1-Ts13 after siRNA treatment against *KLF5*. DLD1-WT reached 50% knockdown while DLD1-Ts13 reached 66% knockdown, resulting in a similar level of absolute protein expression. n=3 technical repeats ***, p=0.0002 and ***, p= 0.0009; One-sample t-test.

B. Representative growth curves of DLD1-WT and DLD1 Ts13 treated with an siRNA against *KLF5* or with a control siRNA. Cell confluence was followed for 72 hrs by live-cell imaging.

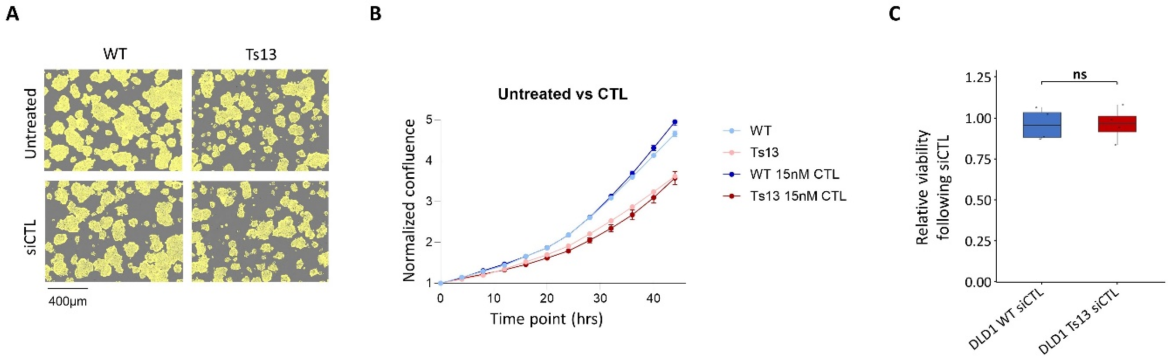

Fig. S14: The introduction of siRNA does not affect the viability of DLD1 isogenic cell lines.

A. Representative images of DLD1-WT and DLD1-Ts13 cells untreated or treated with a control siRNA. Cell masking (shown in yellow) was performed using live cell imaging (IncuCyte) for 72 hrs. Scale bar 400µm.

B. Representative growth curves of DLD1-WT and DLD1-Ts13 with and without 15nM control siRNA. Cell confluence was followed for 72 hrs by live-cell imaging. No difference was observed between untreated cells and those exposed to control siRNA.

C. Quantification of the relative response to 15nM siRNA control treatment between DLD1-WT and DLD1-Ts13, as evaluated by quantifying cell confluence after 72 hrs. n=4 independent experiments. ns, p=0.4925; one-sided paired t-test.

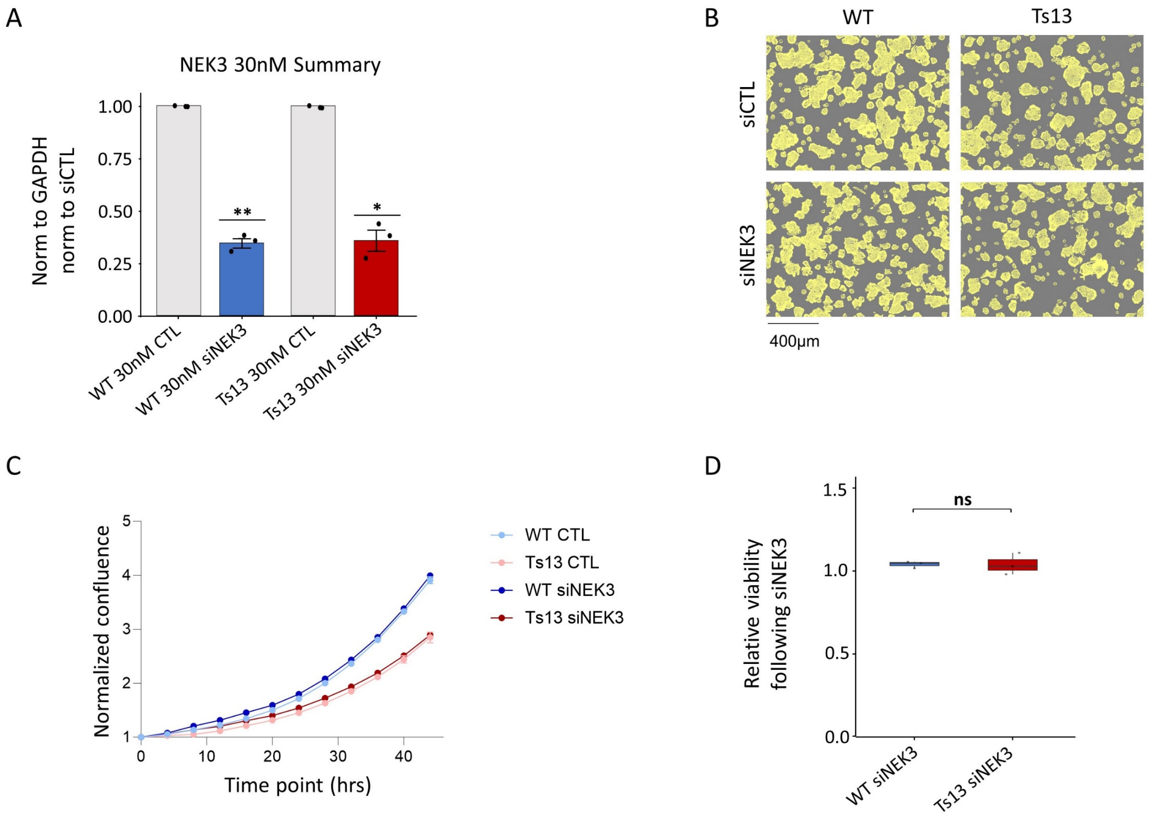

Fig. S15: DLD1 isogenic cell lines exhibit similar responses to *NEK3* knockdown.

A. Comparison of *NEK3* mRNA levels between DLD1-WT and DLD1-Ts13 after treatment with an siRNA against *NEK3*. Both DLD1-WT and DLD1-Ts13 reached efficient knockdown. n=3 independent experiments. **, p=0.0044 and *, p=0.0186; One-sample t-test.

B. Representative images of DLD1-WT and DLD1-Ts13 cells treated with an siRNA against *NEK3*. No proliferation effect was observed following the knockdown. Cell masking (shown in yellow) was performed using live cell imaging (IncuCyte) for 48 hrs. Scale bar 400μm.

C. Representative growth curves of DLD1-WT and DLD1-Ts13 treated with an siRNA against *NEK3* or with a control siRNA. The cell confluence was followed for 48 hrs by live-cell imaging.

D. Quantification of the relative response to *NEK3* knockdown between DLD1-WT and DLD1-Ts13, as evaluated by quantifying cell confluence following a 48 hr treatment with an siRNA against *NEK3* vs. a control siRNA. n=3 independent experiments. ns, p=0.987; two-sided paired t-test.

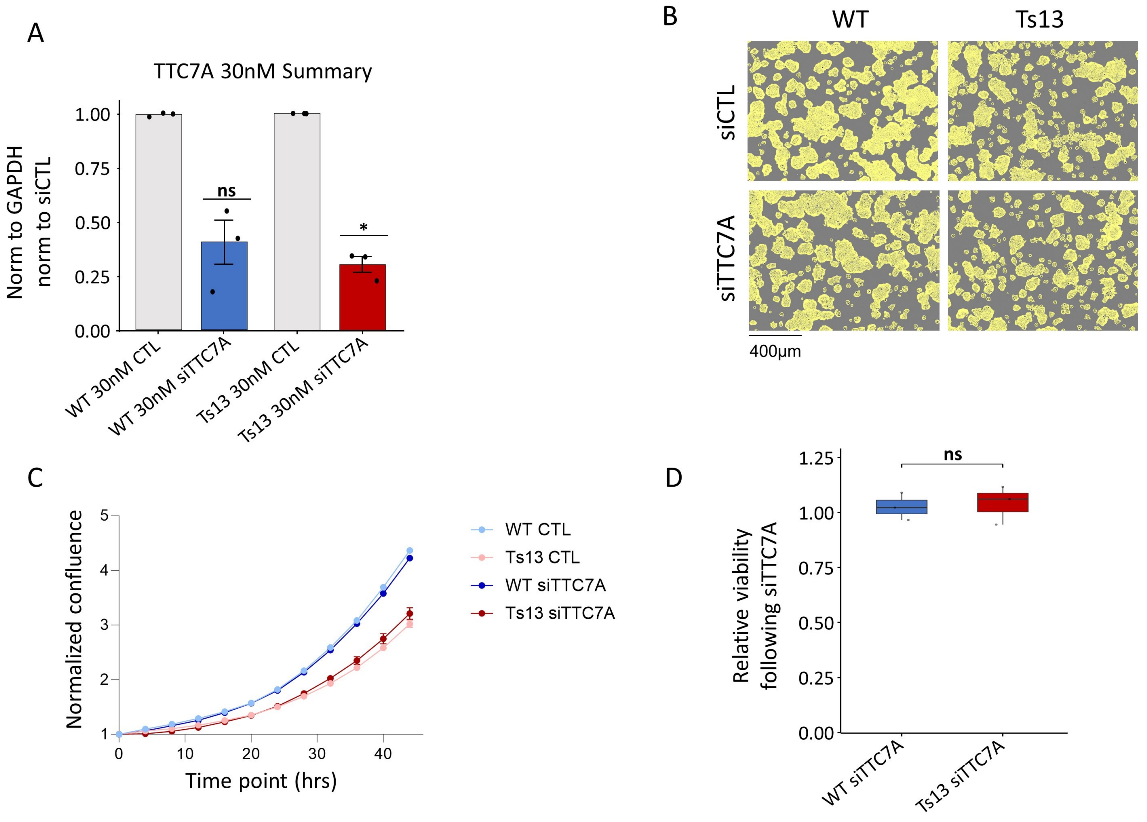

Fig. S16: DLD1 isogenic cell lines exhibit similar responses to *TTC7A* knockdown.

A. Comparison of *TTC7A* mRNA levels between DLD1-WT and DLD1-Ts13 after treatment with an siRNA against *TTC7A*. Both DLD1-WT and DLD1-Ts13 reached efficient knockdown. n=3 independent experiments. ns, p=0.0571 and *, p=0.015; One-sample t-test.

B. Representative images of DLD1-WT and DLD1-Ts13 cells treated with an siRNA against *TTC7A*. No proliferation effect was observed following the knockdown. Cell masking (shown in yellow) was performed using live cell imaging (IncuCyte) for 48 hrs. Scale bar 400µm.

C. Representative growth curves of DLD1-WT and DLD1-Ts13 treated with an siRNA against *TTC7A* or with a control siRNA. The cell confluence was followed for 48 hrs.

D. Quantification of the relative response to *TTC7A* knockdown between DLD1-WT and DLD1-Ts13, as evaluated by quantifying cell confluence following a 48 hr treatment with an siRNA against *TTC7A* vs. a control siRNA. n=3 independent experiments. ns, p=0.796; two-sided paired t-test.

A

	Essential	Intermediate	Non-essential
**Gained paralog**	1,720	1,743	2,155
**Neutral paralog**	2,915	3288	4592
**Lost paralog**	4,392	5,586	8,155

B

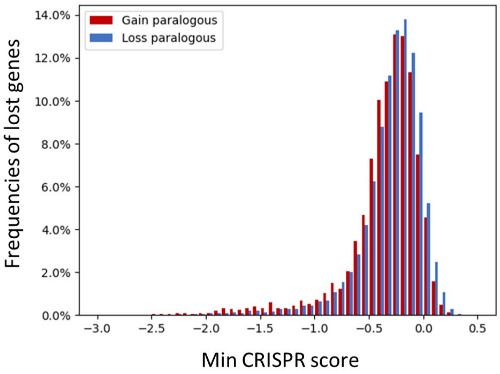

Fig. S17. Support for the role of paralog compensation in shaping tissue-specific aneuploidy patterns. A. Analysis of recurrently-lost genes according to their essentiality and the aneuploidy pattern of their paralog (p=2.38e-24, Chi-squared test).

B. The distribution of recurrently-lost genes by their essentiality (CRISPR score, [3]). Recurrently-lost genes with a frequently-gained paralog tend to be more essential than those with recurrently-lost paralog (p=9.2e-16, KS-test).

References

1. GTEx Consortium. The GTEx Consortium atlas of genetic regulatory effects across human tissues. Science. 2020;369(6509):1318-30.
2. Cardoso-Moreira M, Halbert J, Valloton D, Velten B, Chen C, Shao Y, et al. Gene expression across mammalian organ development. Nature. 2019;571(7766):505-9.
3. Tsherniak A, Vazquez F, Montgomery PG, Weir BA, Kryukov G, Cowley GS, et al. Defining a Cancer Dependency Map. Cell. 2017;170(3):564-76 e16.
