# Supplementary material for: Machine-learning analysis reveals an important role for negative selection in shaping cancer aneuploidy landscapes
Source: Genome Biol. 2024 Apr 15;25:95. doi: 10.1186/s13059-024-03225-7 (PMC11020441; doi:10.1186/s13059-024-03225-7)
Supplement: Supplementary file 8 — Additional file 8. Review history [file 13059_2024_3225_MOESM8_ESM.docx]

**1^st^ round**

**Reviewer 1**

In this manuscript entitled "Machine-learning analysis of factors that shape cancer aneuploidy landscapes reveals an important role for negative selection," Jubran and colleagues used a new machine-learning based-method to determine how different features contribute to selection of individual aneuploidy events in cancer. The findings here nicely complement and explain how different features known to contribute to aneuploidy selection can be prioritized (ie OG/TSG number is most important, but gene expression and essentiality is also important). This work will be of interest to the cancer genomics and larger genomics community as well.
Major Points:
1. Can the authors provide additional explanation (in the results) of how the expression analysis was done? Since there seems to be only one other similar analysis, that suggests to me that it is newer and may not only be confusing to me.
2. The text about paralog compensation (bottom of page 15/top of page 16) needs to be clarified. I misread it a few times, because I didn't understand why deletion and gain would be the opposite direction. I realize now it may be because the compensation is in different directions? However, I misread this to mean that their was compensation in one and not the other.
Minor Points:
1. A more detailed description of Patkar et al may be helpful. I personally had not seen it, and was surprised by the findings.
2. First paragraph of discussion, line 3, I think should be Sack and Davoli as citations (not Taylor).

**Reviewer 2**

Jubran and colleagues present a very interesting manuscript investigating the factors underlying the long-enigmatic tissue-type specific patterns of aneuploidy observed in cancer genomes. The authors use interpretable machine learning methods to examine the relative contribution of a large panel of potential factors to this phenomenon. Interestingly the major factor is revealed to be the force of negative selection, which is often under-considered in cancer genomics partly due to difficulties in measuring and detecting evidence of its contribution to tumour evolution.
Overall I find this manuscript extremely well written, and the figures are well presented. In the main the data and analyses support their conclusions, and the main message is important, timely and interesting for the readership of Genome Biology. I have a few comments particularly focussing on the functional assays regarding KLF5 and chromosome 13q gain. I did not comment on the machine learning approaches since these are outside of my expertise.


1a. The section on validating the unusual gain of 13q in CRC is interesting, but I have a few suggestions that might improve the robustness of this section. The authors explain that 13q is rarely lost in CRC, and often in fact gained. They test the effect of partial depletion of the mRNA for KLF5 in CRC cell lines with disomic, or trisomic 13q, and show a greater sensitivity in the 13q-gained line. Because of the important stated role of this gene in colon development, perhaps the initial most obvious explanation for a gain of this region rather than a loss (seen in many other cancers) is a protection against deleterious loss. However, it is not clear to me why the need to protect against loss of KLF5 would explain the greater sensitivity to its depletion from an 'overexpressed state' in the cell lines? If these lines are more dependent on KLF5 that would suggest a positive effect of higher KLF expression - over and above any negative effect of KLF5 loss. What might this be? Could they comment on this?

1b. I wonder if it may also be important to take into account the absolute levels of knockdown in the two cell lines; mRNA levels suggest similar relative knockdown, but that would leave a different absolute amount of mRNA between the models (knockdown of ~50% from diploid leaves expression of the equivalent of one genomic copy, whereas knockdown from trisomic levels leaves the equivalent of 1.5 copies, in a cell line that is presumably diploid for the rest of the genome, yet is still more sensitive).

1c. Additionally - it would strengthen their conclusions here to examine the relative sensitivity of the DLD1 Ts13 cells to depletion of other genes in addition to KLF5 - perhaps some controls related to similar genes to KLF5 that do not reside on 13q, or perhaps some genes that do reside on 13q apart from KLF5? For example, these cell lines may have become dependent upon overexpression of many genes on 13q, independent of a connection to the initial selection pressure driving the original gain of this region.

1d. Could we also see the absolute decrease in viability for siControl conditions in addition to siKLF5 in the graphs. The images hint that the DLD1 ts13 cells might be more sensitive to siRNA-mediated depletion in general, which might need to be taken into account when interpreting these results.

2. Their results are analysed in terms of general, widespread contributions to aneuploidy patterns across many cancer types and all chromosome arms. It would be very interesting to also present, or provide the data in terms of individual chromosome-arm:tumour type comparisons, as contributions might vary across these, yielding important information for the field.
3. Relatedly, Is it possible to pick out the chromosome-arm:tumour type pairs that are the strongest drivers of the most important features for the ML model?For example, might this general effect be driven by a subset of chromosome-tumour interactions?

Minor points

4. Can graphs Fig 4 I,j have y-axis grounded in zero for easier comparison?


5. Figure 4a,c and e: perhaps the axis label could read 'colorectal cancer cells', rather than 'colorectal cells', to avoid confusion.

**Reviewer 3**

Summary:
In the manuscript "Machine-learning analysis of factors that shape cancer aneuploidy landscapes reveals an important role for negative selection" (GBIO-D-23-00966) the authors used interpretable machine learning analysis to identify features associated with aneuploidy. Two machine learning models were developed, one model analyzed chromosome-arm gain and the other chromosome-arm loss. The models had different top features and their results appear to be in line with other works in the field. Overall, the paper is well written with an interesting premise and novel conclusions. However, it does require some revision to the main text in order to maximize clarity and address missing topics.

Major concerns:
1. The discussion regarding Chromosome 13q aneuploidy is not clear whether the results are in reference to a particular colorectal cancer cell line (presumably derived from CCLE/depmap) or a primary colorectal cancer derived from TCGA, or both - the phrase "colorectal cancer cell line" is used interchangeably with "colorectal cancer". Please clarify. If these results are in reference to a particular cell line, this cell line should be named here, to match the name in CCLE, and the specific colorectal cancer from which it is derived should also be named. If it is in reference to a TCGA cancer (one of the 24 cancers featured in figure 1A y axis), then "colorectal cancer" should probably be modified to colorectal adenocarcinoma (COAD), to match - though based on figure 4, it does not seem that primary human cancer data was used for these conclusions, so this is likely not appropriate.
a. If all the results regarding this chr13q pattern are in reference to a particular colorectal cell line, then throughout the paper, all references to "colorectal cancer" should be replaced with the name of the cell line - it is unclear whether it is appropriate to make any conclusions about primary colorectal cancer based on analysis derived from an isolated cell line. For example, the title of Figure 4 should be modified to read "KLF5 is a potential driver of chromosome 13q gain in [name of cell line]". Previous research has shown that not all cell lines are equal in their ability to model primary human tumors: https://www.nature.com/articles/s41467-019-11415-2#Sec8
b. Considering that the authors have access to data from primary COAD from TCGA as evidenced by figure 1A, why not see if these results for chr13q are also true for this data? If there is room to explore this topic within this study, it could be interesting - otherwise, could be left for future endeavors.
2. This paper should clarify that its focus is on gain/loss patterns partial aneuploidies (single arm gains and losses). It does not include discussion of other kinds of aneuploidies such as monosomies, disomies, trisomies, tetrasomies, aside from an investigation of the DLD1 cell line which has trisomy 13 - if these additional types of aneuploidy can be addressed within the current paper, this would be ideal - otherwise please describe how the current analysis could be expanded to these other types of aneuploidy in future studies.

Minor concerns:
1. Please include some reference to mosaicism in cancer aneuploidies - if this topic cannot be addressed with the available data from TCGA/GTEx/CCLE, please describe how it could be investigated with future endeavors
2. On Page 21 and lines 51-52, how can a lower q-value be chosen when there is a tie? In the case of a tie, why are both q-values not equal?
3. How is the height of each feature interpreted in Figure 2 panels B and D?
4. For clarity, please combine these two statements in the abstract, so it is clear to a reader that a key finding in this paper is that negative selection is driving the identification of gain/loss patterns via TSG/OG density analysis: "While confirming known drivers of positive selection, our quantitative analysis highlighted the importance of negative selection for shaping the aneuploidy landscapes of human cancer. Tumor suppressor gene density was a better predictor of gain patterns than oncogene density, and vice-versa for loss patterns."
5. Abbreviations in the results section were defined in the methods section rather than where the abbreviation was first encountered in the manuscript (for example, GISTIC2).
6. The in-text reference for GTEx consortium is interchangeably called "Consortium 2020" and "GTEx Consortium 2020" - please modify this for consistency (use the latter). Also, for clarity, please ensure that GTEx is first mentioned side-by-side to TCGA in the first paragraph of the results section, where normal tissue-of-origin data is first discussed. It is somewhat odd to read about normal tissue expression data in the subsequent paragraph without the paper having referenced where it was obtained from.
7. In a similar vein to the previous item, please mention Depmap/Cancer Cell Line Encylcopedia when this data is first discussed, under the header "Similar predictive features in human cancer cell lines and in human tumors".
8. Please revise this sentence for clarity, as it is quite confusing: "In human cancer, a chromosome-arm is either recurrently gained or recurrently lost across cancer types, but is only rarely gained in some cancer types and lost in others"
9. On page 8 line 21, the authors mention 'five different ML methods' are used for this research, but none are listed by name. Please refer to the Methods section here.
10. Please add a download date and/or version number of the TCGA data used in this study.

**Reviewer 4**

Jubran et al an ML-based analysis of the aneuploidies observed in TCGA and CCLs. Presentation is clear except for the need for clarification of the ML model features. The experimental findings seem to support the findings about paralog compensation mechanisms but multiple points should be clarified about how to define and measure essentiality in the presence of paralogs.

It is also useful to compare the study with previous methods and analyses on chromosome-level copy number analysis.

I include all my comments below, which i hope will be useful for improving the manuscript:

1. Please explain how the current study is different from the other studies that analyzed copy number patterns in cancers, including TCGA.

2. "prominent examples are the tumor suppressors TP53 and PTEN, which have been shown to drive the recurrent loss of chromosome-arm 17p in leukemia and that of 10p in glioma, respectively (Liuet al. 2016; Zhou et al. 1999)"
This sentence seems to suggest that PTEN mutations cause loss of 10p in glioma, can the authors please provide supporting evidence from literature? I could not find it in the paper or in pubmed. My understanding is that gliomas are generally characterized by 10q abnormalities.

3. "Therefore, whereas positive selection greatly outweighs negative selection in shaping the landscape of point mutations in cancer (Martincorena et al. 2017)"
Please provide the metric with respect to which we can see that positive selection outweighs negative selection in point mutation landscape.

4. Please explain how a chromosome-arm event is defined. Is it on a length basis? For example, if a copy number that comprises 70% of the chromosome arm, does that count as a arm-level event?

5. Please provide how the features are correlated with each other and explain how that may lead affect the current study. Also, it would be useful to visualize the all arm-level aneuploidies in pca, tsne, etc.

6. Interpreting SHAP results is hard since study build 2 separate models and calculates SHAP independently because model a binary classification. Is it possible to do a 3-class classification to distinguish all of the events? I feel like this would be more appropriate to interpret SHAP results.

7. One issue is that the aneuploidies do not happen in isoluation, i.e., a patient may have multiple aneuploidies or smaller events on other chromosomes. The model seems to treat each arm-level event separately and the information regarding co-incidence of arm-level event is lost. It would be useful to know the correlations between the events used in this study. This could have an impact on the trained model.

8. Please provide results about the robustness of the model with respect to internal parameters (e.g. 10% cutoff for gene-based features.) to generate the features and the hyperparameters of the ML models.

9. It is not clear how the origin tissue is determined for all cancers from GTEx. For example, for blood cancers, did authors use whole blood? Similarly for brain tumors which tissue was selected? How much does this change final accuracy?

10. There are numerous methods that estimate impact of structural variants on a variant-level basis. Please explain how to distinguish the current study from the other methods in terms of findings and technical approach.

11. SHAP has numerous known limitations that should be discussed in more detail.

12. The details of the essentiality analysis of UCHL3 is missing (e.g. Fig 5c). Please describe how the x and y axies are calculated in figures presenting scatter plots of essentiality scores and dependencies.

13. I think the transition of the manuscript from ML-model to the focus on paralog compensation a little abrupt. While there are 20 features, only a handful of them were discussed in terms of importance. Also, it is not clear why authors chose to focus on paralog compensation as the major feature to follow up on with experimental validations. This feature was not the most important feature among other features and was not presented among the top features for gain model. For example PPIs seems to have more importance in both models but they were not studied more extensively in the manuscript.

14. I find paralog compensation features to be important, however it is not clear how they correlate with other features. For example, gene expression levels of paralogs may not be reliably estimated (e.g., read mapping) and may impact the interpretability of the model.

15. I find the definition of essentiality on paralogs a little confusing. If a gene has a paralog, it should by definition not be an essential gene. The exact definition should be clarified in the study.

16. Also, the reliability of crispr-cas9 system to quantify the essentiality of a gene with multiple paralogs may be biased.

17. Fix Parenthesis: '...the gene was neutral (Taylor et al. study (Taylor et al. 2018). Essentiality by CRISPR..."

**Authors’ response**

Reviewer #1:

In this manuscript entitled "Machine-learning analysis of factors that shape cancer aneuploidy landscapes reveals an important role for negative selection," Jubran and colleagues used a new machine-learning based-method to determine how different features contribute to selection of individual aneuploidy events in cancer. The findings here nicely complement and explain how different features known to contribute to aneuploidy selection can be prioritized (ie OG/TSG number is most important, but gene expression and essentiality is also important). This work will be of interest to the cancer genomics and larger genomics community as well.

Major Points:

1. Can the authors provide additional explanation (in the results) of how the expression analysis was done? Since there seems to be only one other similar analysis, that suggests to me that it is newer and may not only be confusing to me.

We revised the Results to explain better the computation of expression-related features. Second paragraph in the Results section, page 5.

2. The text about paralog compensation (bottom of page 15/top of page 16) needs to be clarified. I misread it a few times, because I didn't understand why deletion and gain would be the opposite direction. I realize now it may be because the compensation is in different directions? However, I misread this to mean that their was compensation in one and not the other.

We clarified this part by focusing only on paralog compensation and deletion (chromosome-arm loss). We removed the analysis of paralog compensation in the gain model since paralog compensation was not among its top contributing features. Please see Results section (page 13, second paragraph).

Minor Points:

1. A more detailed description of Patkar et al may be helpful. I personally had not seen it, and was surprised by the findings.

We added a description of Patkar et al. in the Introduction section, page 3, second paragraph.

2. First paragraph of discussion, line 3, I think should be Sack and Davoli as citations (not Taylor).

The Reviewer is correct, this reference has been fixed, thank you.

Reviewer #2:

Jubran and colleagues present a very interesting manuscript investigating the factors underlying the long-enigmatic tissue-type specific patterns of aneuploidy observed in cancer genomes. The authors use interpretable machine learning methods to examine the relative contribution of a large panel of potential factors to this phenomenon. Interestingly the major factor is revealed to be the force of negative selection, which is often under-considered in cancer genomics partly due to difficulties in measuring and detecting evidence of its contribution to tumour evolution.

Overall I find this manuscript extremely well written, and the figures are well presented. In the main the data and analyses support their conclusions, and the main message is important, timely and interesting for the readership of Genome Biology. I have a few comments particularly focusing on the functional assays regarding KLF5 and chromosome 13q gain. I did not comment on the machine learning approaches since these are outside of my expertise.

1a. The section on validating the unusual gain of 13q in CRC is interesting, but I have a few suggestions that might improve the robustness of this section. The authors explain that 13q is rarely lost in CRC, and often in fact gained. They test the effect of partial depletion of the mRNA for KLF5 in CRC cell lines with disomic, or trisomic 13q, and show a greater sensitivity in the 13q-gained line. Because of the important stated role of this gene in colon development, perhaps the initial most obvious explanation for a gain of this region rather than a loss (seen in many other cancers) is a protection against deleterious loss. However, it is not clear to me why the need to protect against loss of KLF5 would explain the greater sensitivity to its depletion from an 'overexpressed state' in the cell lines? If these lines are more dependent on KLF5 that would suggest a positive effect of higher KLF expression - over and above any negative effect of KLF5 loss. What might this be? Could they comment on this?

This is a valid point, in line with KLF5 having an oncogenic role in CRC. We modified the text in the Discussion to explain that indeed the findings hint at a positive selection for KLF5 gain, beyond the negative selection against its loss. Please see the Discussion section (page 17, third paragraph).

1b. I wonder if it may also be important to take into account the absolute levels of knockdown in the two cell lines; mRNA levels suggest similar relative knockdown, but that would leave a different absolute amount of mRNA between the models (knockdown of ~50% from diploid leaves expression of the equivalent of one genomic copy, whereas knockdown from trisomic levels leaves the equivalent of 1.5 copies, in a cell line that is presumably diploid for the rest of the genome, yet is still more sensitive).

Thank you for this suggestion. As suggested, we reached an absolute knockdown of KLF5 in both cell lines by knocking down ~50% KLF5 mRNA levels in DLD1-WT and ~66% KLF5 mRNA levels in the DLD1-Ts13 cells. We then followed cell proliferation via live cell imaging to assess the effect of knockdown to a similar absolute levels KLF5. Indeed, we found that under these conditions, the DLD1-Ts13 cells were still more sensitive to KLF5 knockdown in comparison to the DLD-WT cells. Please see the new Fig.S13 and the Results section (page 12, first paragraph).

1c. Additionally - it would strengthen their conclusions here to examine the relative sensitivity of the DLD1 Ts13 cells to depletion of other genes in addition to KLF5 - perhaps some controls related to similar genes to KLF5 that do not reside on 13q, or perhaps some genes that do reside on 13q apart from KLF5? For example, these cell lines may have become dependent upon overexpression of many genes on 13q, independent of a connection to the initial selection pressure driving the original gain of this region.

This is indeed an interesting suggestion. We followed up on it by testing two additional genes that can serve as distinct controls. The first control, NEK3, resides on chromosome 13q, is expressed in colon, and has a completely different cellular role from KLF5 (NEK3 is a serine-threonine kinase that is involved in cytoskeletal organization and motility). As a second control, we considered TTC7A, a gene that is located on another chromosome (2p), is expressed in the colon, and has a similar function to KLF5 (TTC7A is a transcription factor involved in the development of the intestinal tract).

We individually knocked down each of these genes to a comparable level of knockdown as we originally obtained with KLF5 (~70%). We found that neither the knockdown of NEK3 nor that of TTC7A had any differential effect on the proliferation and viability of the cells. We therefore conclude that the increased sensitivity of DLD1- Ts13 cells to KD of KLF5 is not shared by all chr13q-residing genes, nor is it shared by all genes with a similar role to that of KLF5. Please see the new Fig.S15 and Fig.S16 and the Results section (page 12, first paragraph).

In addition, to confirm that DLD1-Ts13 are not generally more sensitive than DLD1-WT to toxic perturbations, we treated the cells with two cytotoxic drugs with different mechanisms of action: the DNA damage inducer etoposide, and the proteasome inhibitor bortezomib. No significant difference was observed between the cells’ response to these drugs (Figure 1 below).

Figure 1: Dose response plots of DLD1-WT and DLD1-Ts3 cells to two cytotoxic drugs. Cell were treated with the drugs (at the mentioned concentrations) for 72hr.

1d. Could we also see the absolute decrease in viability for siControl conditions in addition to siKLF5 in the graphs. The images hint that the DLD1 ts13 cells might be more sensitive to siRNA-mediated depletion in general, which might need to be taken into account when interpreting these results.

The difference between the growth of the DLD1-WT and Ts cells, which was evident in the images, is actually not due to a differential response to the siControl oligos, but rather due to the slower proliferation rate of the aneuploid cells under standard culture conditions. DLD1-Ts13 cells proliferate slower than the DLD1-WT cells, as was previously reported by Ruthledge et al. 2016, Scientific Reports. We mention this difference in proliferation rate explicitly – please see the legend to Fig. 4I. Importantly, the results from each cell line are normalized against its respective control. To rule out siRNA toxicity, we compared DLD1 isogenic cells with and without siControl treatment. We assessed cell confluence using live cell imaging and observed no discernible difference in response to siRNA treatment between DLD1-WT and DLD1-Ts13 cells. Please see the new Fig.S14 and the Results section (page 12, first paragraph). We also replaced the images in Fig. 4I to prevent confusion – please see the revised Fig.4.

2. Their results are analysed in terms of general, widespread contributions to aneuploidy patterns across many cancer types and all chromosome arms. It would be very interesting to also present, or provide the data in terms of individual chromosome- arm:tumour type comparisons, as contributions might vary across these, yielding important information for the field.

Thank you for this interesting comment. We have now added a new supplementary table that provides the data in terms of individual chromosome-arm:tumour type pairs. Specifically, Table S3 details, for each pair, its contribution (SHAP value) per feature to the gain model and to the loss model (Table S3). Also see our response to comment 3 below.

3. Relatedly, Is it possible to pick out the chromosome-arm:tumour type pairs that are the strongest drivers of the most important features for the ML model? For example, might this general effect be driven by a subset of chromosome-tumour interactions?

To pick out the chromosome-arm:tumour type pairs that are strong drivers of each feature, we used their SHAP values. We defined a pair as a strong driver if its SHAP value was: (i) among the top 10% most positive feature values, suggesting it was a potential driver of that gain/loss event; or (ii) among the 10% most negative values for that feature, suggesting it was acting against that gain/loss. We provide this information in a new Table S4.

To test if the general effects we observed were driven by a subset of chromosome- tumor interactions, we focused on chromosome-arm:tumour type pairs that were strong drivers of the most important features of each model. Next, we re-created the gain and loss models upon excluding those pairs. We found that the top-contributing features to each model and their directionality were generally retained. This analysis was added to the manuscript in the Results: page 9, second paragraph; Discussion: page 15, first paragraph; and Methods: page 23, second paragraph.

Minor points

4. Can graphs Fig 4 I,j have y-axis grounded in zero for easier comparison?

Yes, we modified the y-axis of these plots accordingly.

5. Figure 4a,c and e: perhaps the axis label could read 'colorectal cancer cells', rather than 'colorectal cells', to avoid confusion.

Yes, we modified the labeling. We also added data from colorectal tumors and added them to these Figure panels (see below our response to Reviewer #3), and so the axis labels have been changed to reflect when we refer to tumors and when to cell lines.

Reviewer #3:

In the manuscript "Machine-learning analysis of factors that shape cancer aneuploidy landscapes reveals an important role for negative selection" (GBIO-D-23-00966) the authors used interpretable machine learning analysis to identify features associated with aneuploidy. Two machine learning models were developed, one model analyzed chromosome-arm gain and the other chromosome-arm loss. The models had different top features and their results appear to be in line with other works in the field. Overall, the paper is well written with an interesting premise and novel conclusions. However, it does require some revision to the main text in order to maximize clarity and address missing topics.

Major concerns:

1. The discussion regarding Chromosome 13q aneuploidy is not clear whether the results are in reference to a particular colorectal cancer cell line (presumably derived from CCLE/depmap) or a primary colorectal cancer derived from TCGA, or both - the phrase "colorectal cancer cell line" is used interchangeably with "colorectal cancer". Please clarify. If these results are in reference to a particular cell line, this cell line should be named here, to match the name in CCLE, and the specific colorectal cancer from which it is derived should also be named. If it is in reference to a TCGA cancer (one of the 24 cancers featured in figure 1A y axis), then "colorectal cancer" should probably be modified to colorectal adenocarcinoma (COAD), to match - though based on figure 4, it does not seem that primary human cancer data was used for these conclusions, so this is likely not appropriate.

We apologize for this misunderstanding. The results originally shown in Fig. 4 were based on the combined analysis of (n=85) CRC cell lines, not to any particular cell line. Based on the Reviewer’s request below, we have recapitulated this analysis in (n=434) TCGA COAD tumors, and we have added these results to Figure 4. The main text has been clarified accordingly.

a. If all the results regarding this chr13q pattern are in reference to a particular colorectal cell line, then throughout the paper, all references to "colorectal cancer" should be replaced with the name of the cell line - it is unclear whether it is appropriate to make any conclusions about primary colorectal cancer based on analysis derived from an isolated cell line. For example, the title of Figure 4 should be modified to read "KLF5 is a potential driver of chromosome 13q gain in [name of cell line]". Previous research has shown that not all cell lines are equal in their ability to model primary human tumors: https://imsva91- ctp.trendmicro.com:443/wis/clicktime/v1/query?url=https%3a%2f%2fwww.nature.com %2farticles%2fs41467%2d019%2d11415%2d2%23Sec8&umid=7B67EAC3-064A- 3B06-A85A-B593AC5E9AD4&auth=701951cf8dfb7cd99762e0f5be252082d875f1cf- 5cefa24a4f1a276d2448adb845870d4f85f5a5cb

All of the results pertain to a combined analysis of (n=85) colorectal cell lines, and in the revised manuscript they also pertain to a combined analysis (n=434) of colorectal adenocarcinoma tumors. It is only the in vitro experiments shown in Fig. 4H-J that were performed in one CRC cell line – the isogenic DLD1 cell line with/without trisomy of chr13. This is now clearly presented in the main text, in the legend and on the plots themselves.

b. Considering that the authors have access to data from primary COAD from TCGA as evidenced by figure 1A, why not see if these results for chr13q are also true for this data? If there is room to explore this topic within this study, it could be interesting - otherwise, could be left for future endeavors.

We thank the Reviewer for this excellent suggestion. We have reproduced the cell line analyses with primary tumor data from the TCGA COAD cohort. Indeed, chr13q was an outlier in COAD tumors, and KLF5 was among the top differentially expressed genes in COAD. Please see the revised Fig. 4A, 4B, 4E, and 4F and the section “Chromosome 13q aneuploidy” of the Results.

2. This paper should clarify that its focus is on gain/loss patterns partial aneuploidies (single arm gains and losses). It does not include discussion of other kinds of aneuploidies such as monosomies, disomies, trisomies, tetrasomies, aside from an investigation of the DLD1 cell line which has trisomy 13 - if these additional types of aneuploidy can be addressed within the current paper, this would be ideal - otherwise please describe how the current analysis could be expanded to these other types of aneuploidy in future studies.

Following the Reviewer’s suggestion, we expanded our analyses to address whole- chromosome monosomies and trisomies. These analyses were added to the Results: page 9, third paragraph, and Fig. S11; Discussion: page 15, first paragraph, and page 16, first paragraph; and Methods: page 18, last paragraph. We also added to the Discussion a description of how the current analysis could be expanded to additional types of aneuploidy (e.g., tetrasomies) in future studies (page 17, second paragraph).

Minor concerns:

1. Please include some reference to mosaicism in cancer aneuploidies - if this topic cannot be addressed with the available data from TCGA/GTEx/CCLE, please describe how it could be investigated with future endeavors.

We introduced a short discussion of this important topic to the Discussion section. Please see page 17, second paragraph.

2. On Page 21 and lines 51-52, how can a lower q-value be chosen when there is a tie? In the case of a tie, why are both q-values not equal?

This mistake was fixed, thank you for noticing this inaccuracy. It was revised in the Methods (“Chromosome-arm aneuploidy patterns per cancer” section, page 18 and “Chromosome-arm aneuploidy patterns per CCLs” section, page 24).

3. How is the height of each feature interpreted in Figure 2 panels B and D?

The height of each interpreted feature corresponds to the relative contribution of that feature to the model, as calculated by SHAP package. We revised the legend of Fig. 2B and the Methods (“SHAP analysis of feature importance” section, page 22).

4. For clarity, please combine these two statements in the abstract, so it is clear to a reader that a key finding in this paper is that negative selection is driving the identification of gain/loss patterns via TSG/OG density analysis: "While confirming known drivers of positive selection, our quantitative analysis highlighted the importance of negative selection for shaping the aneuploidy landscapes of human cancer. Tumor suppressor gene density was a better predictor of gain patterns than oncogene density, and vice-versa for loss patterns."

We connected these two sentences by explicitly stating that the gene density is a notable example of negative selection. Please see the revised Abstract.

5. Abbreviations in the results section were defined in the methods section rather than where the abbreviation was first encountered in the manuscript (for example, GISTIC2).

This was fixed, thank you for spotting (page 5, second paragraph).

6. The in-text reference for GTEx consortium is interchangeably called "Consortium 2020" and "GTEx Consortium 2020" - please modify this for consistency (use the latter). Also, for clarity, please ensure that GTEx is first mentioned side-by-side to TCGA in the first paragraph of the results section, where normal tissue-of-origin data is first discussed. It is somewhat odd to read about normal tissue expression data in the subsequent paragraph without the paper having referenced where it was obtained from.

This in-text reference for GTEx consortium was fixed, and GTEx appears where normal tissue-of-origin data is first discussed (page 5, second paragraph).

7. In a similar vein to the previous item, please mention Depmap/Cancer Cell Line Encylcopedia when this data is first discussed, under the header "Similar predictive features in human cancer cell lines and in human tumors".

This was fixed Results: page 5, third paragraph; Methods: page 19, last paragraph, thank you.

8. Please revise this sentence for clarity, as it is quite confusing: "In human cancer, a chromosome-arm is either recurrently gained or recurrently lost across cancer types, but is only rarely gained in some cancer types and lost in others".

We revised this sentence for clarity. (page 11, “Chromosome 13q aneuploidy” section, first paragraph).

9. On page 8 line 21, the authors mention 'five different ML methods' are used for this research, but none are listed by name. Please refer to the Methods section here.

The text now refers to the Methods, as requested (page 6, third paragraph).

10. Please add a download date and/or version number of the TCGA data used in this study.

The download date and version number have been added. Please see Methods, page 19, “Features of cancer tissues” section.

Reviewer #4:

Jubran et al an ML-based analysis of the aneuploidies observed in TCGA and CCLs. Presentation is clear except for the need for clarification of the ML model features. The experimental findings seem to support the findings about paralog compensation mechanisms but multiple points should be clarified about how to define and measure essentiality in the presence of paralogs. It is also useful to compare the study with previous methods and analyses on chromosome-level copy number analysis.

I include all my comments below, which i hope will be useful for improving the manuscript:

1. Please explain how the current study is different from the other studies that analyzed copy number patterns in cancers, including TCGA.

We edited the Discussion to explain how the current study is different from other studies that analyzed copy number patterns in cancers. Please see the first paragraph of the Discussion, page 14.

2. "prominent examples are the tumor suppressors TP53 and PTEN, which have been shown to drive the recurrent loss of chromosome-arm 17p in leukemia and that of 10p in glioma, respectively (Liuet al. 2016; Zhou et al. 1999)"

This sentence seems to suggest that PTEN mutations cause loss of 10p in glioma, can the authors please provide supporting evidence from literature? I could not find it in the paper or in pubmed. My understanding is that gliomas are generally characterized by 10q abnormalities.

The Reviewer is correct, this was a mistake, PTEN is on 10q not on 10p. This mistake was corrected, and we now cite papers that consider PTEN as a driver underlying 10q loss in gliomas. Please see the second paragraph of the Introduction, page 3.

3. "Therefore, whereas positive selection greatly outweighs negative selection in shaping the landscape of point mutations in cancer (Martincorena et al. 2017)"

Please provide the metric with respect to which we can see that positive selection outweighs negative selection in point mutation landscape.

Martinocorena et al. estimate that 1-3.9% of all genes are affected by positive selection, leading to 1-10+ mutations per tumor, vs. 0.02-0.5% of all genes affected by negative selection, leading to ~0.5% coding mutations lost per tumor. They also estimate that nearly all (~99%) coding mutations are tolerated and escape negative selection. The metric that is used in their paper to quantitatively assess positive and negative selection is a refined version of dN/dS, the normalized ratio of non- synonymous to synonymous mutations. This is now mentioned in the revised sentence. Please see page 4, first paragraph.

4. Please explain how a chromosome-arm event is defined. Is it on a length basis? For example, if a copy number that comprises 70% of the chromosome arm, does that count as a arm-level event?

Chromosome-arm events were defined according to GISTIC2.0, a probabilistic method for identifying genomic regions that are significantly gained or lost across a set of tumors (ref). This method calculated the significance of chromosome-arm events by comparing the observed frequency of chromosome-arm events to the expected rate, while taking into account chromosome-arm length among other parameters (Beroukhim et al., PNAS 2007). We added this clarification and additional details to the Methods (‘Chromosome-arm aneuploidy patterns per cancer’ section, page 18).

5. Please provide how the features are correlated with each other and explain how that may lead affect the current study. Also, it would be useful to visualize the all arm-level aneuploidies in pca, tsne, etc.

Thank you for these suggestions. Following this comment, we analyzed the correlations between features and added a PCA visualization of all arm-level aneuploidies. The results were described and discussed in the Results: second paragraph, page 6 and Fig. S3; Discussion: page 15, first paragraph; and Methods: “Construction of the final dataset”, page 21.

6. Interpreting SHAP results is hard since study build 2 separate models and calculates SHAP independently because model a binary classification. Is it possible to do a 3- class classification to distinguish all of the events? I feel like this would be more appropriate to interpret SHAP results.

In a limited dataset such as ours, which is not amenable to neural network methods, a 3-class classification is typically implemented in one of two ways: (i) using binary classifiers that compare every two classes, or (ii) using binary classifiers the compare one class versus rest. Performance is then averaged over the different binary classifiers. Notably, in our study we used both: In the main text we presented binary classifiers comparing gain versus neutrality (gain model) and loss versus neutrality (loss model). In the Supplementary material, we presented binary classifiers comparing gain versus rest and loss versus rest (Fig. S9). We showed that in both analyses, interpretation led to similar results. Hence, there was no clear advantage to applying a 3-class classification.

7. One issue is that the aneuploidies do not happen in isolation, i.e., a patient may have multiple aneuploidies or smaller events on other chromosomes. The model seems to treat each arm-level event separately and the information regarding co- incidence of arm-level event is lost. It would be useful to know the correlations between the events used in this study. This could have an impact on the trained model.

Indeed, cancer aneuploidies can co-occur or be mutually-exclusive. We previously reported the co-occurrence patterns of tumors across the TCGA data sets (Prasad et al. Cancer Res 2022). Here, we analyzed the co-occurrence probabilities of chromosome-arm events per cancer. The median fraction of chromosome-arm pairs with significant co-incidence per cancer was 2.05% (Table S6). Hence, the impact of co-incidence on the models is expected to be small. We have added this analysis to the Methods (“Chromosome-arm aneuploidy patterns per cancer” section, page 18 and Table S6) and to the Discussion (page 7, second paragraph).

8. Please provide results about the robustness of the model with respect to internal parameters (e.g. 10% cutoff for gene-based features.) to generate the features and the hyperparameters of the ML models.

We have now assessed the robustness of the model with respect to internal parameters used to generate the features, and the hyperparameters of the ML models. We added these analyses to the Results: “The robust impact of top” section, page 8 and Table S2, Fig. S7, Fig. S8); Discussion: page 15, first paragraph; and Methods: “Robustness analyses” section, page 22.

9. It is not clear how the origin tissue is determined for all cancers from GTEx. For example, for blood cancers, did authors use whole blood? Similarly for brain tumors which tissue was selected? How much does this change final accuracy?

The association of cancers with normal tissues from GTEx is presented in Table S1. Blood cancers were indeed associated with whole blood and brain cancers were associated with brain tissue (see Methods for more information on brain, “Construction of the final dataset”, page 21). Notably, the association was similar to that performed by Patkar et al., Genome Medicine 2021. This is now better clarified in the text. Please see Results section, page 5, last paragraph.

10. There are numerous methods that estimate impact of structural variants on a variant-level basis. Please explain how to distinguish the current study from the other methods in terms of findings and technical approach.

In the current study, we are not looking into structural variants but rather into a numerical variant (copy number changes of entire chromosomes or chromosome arms). As detailed in our response to comment #1, our study is different from previous studies that analyzed copy number patterns in cancer, and a discussion of these differences has been added to the first paragraph of the Discussion section, page 14.

11. SHAP has numerous known limitations that should be discussed in more detail.

We expanded the discussion of SHAP limitations in Discussion, page 15, first paragraph.

12. The details of the essentiality analysis of UCHL3 is missing (e.g. Fig 5c). Please describe how the x and y axies are calculated in figures presenting scatter plots of essentiality scores and dependencies.

In Fig. 4C: These results are based on the Chronos dependency scores of the DepMap CRISPR/Cas9 screens. The x-axis presents the effect size (i.e., the difference between average response values between colorectal cell lines and other cell lines), and the y- axis presents the significance of the difference (-log10(p-value)).

In Fig. 5C: These results are based on the Chronos dependency scores of the DepMap CRISPR/Cas9 screens. The x-axis presents the effect size (i.e., the difference between average response values between chr1q-WT and chr13q-gain colorectal cell lines) and the y-axis (Dempster et al. 2021) presents the significance of the difference (-log10(p- value)).

The legends for both of these plots have been modified to describe the axes, and a reference to the Chronos scores has been added.

13. I think the transition of the manuscript from ML-model to the focus on paralog compensation a little abrupt. While there are 20 features, only a handful of them were discussed in terms of importance. Also, it is not clear why authors chose to focus on paralog compensation as the major feature to follow up on with experimental validations. This feature was not the most important feature among other features and was not presented among the top features for gain model. For example PPIs seems to have more importance in both models but they were not studied more extensively in the manuscript.

In the manuscript we mainly discussed the features that had the highest contribution (importance) to the different models. PPIs features are also interesting and remain to be studied in a future study. However, we chose to focus on paralog compensation as the major feature to follow up on experimentally, since it was a novel feature that was found to be important in loss models (including chromosome-arm loss model in primary tumors and in CCLs, and also in the whole-chromosome loss model), and was easier to test experimentally. Following this comment, we expanded the transition of the manuscript from ML models to the focus on paralog compensation (Results, “paralogs compensation is an important feature” section, second paragraph, page 12), and suggested to investigate the role of PPIs in future studies (Discussion section, page 17, second paragraph).

14. I find paralog compensation features to be important, however, it is not clear how they correlate with other features. For example, gene expression levels of paralogs may not be reliably estimated (e.g., read mapping) and may impact the interpretability of the model.

Indeed, the estimation of gene expression levels of pairs of paralogs could be problematic when the sequence similarity between paralogs is very high. However, the median sequence similarity between paralogs in our dataset was 60% (Figure 2 below). Moreover, the expression datasets that we used (GTEx) estimated gene expression levels by using only uniquely mapped reads. We assessed the correlation between paralogs compensation and other features (Fig. S3A). Paralogs compensation did not correlate positively with any feature, and was most negatively correlated with ‘expression during childhood development’ (Spearman r=-0.7), ‘development expression variation’ (r=-0.6), and ‘tissue-specific gene density’ (r=-0.69), all of which did not rank high by our models. We therefore conclude that this feature was not confounded by any other strong feature that ranked high in our models. Please see also see our response to point #5 above.

Figure 2. The sequence identity between all pairs of paralogs in our dataset.

15. I find the definition of essentiality on paralogs a little confusing. If a gene has a paralog, it should by definition not be an essential gene. The exact definition should be clarified in the study.

Thanks for this comment. We clarified the issue of essentiality of paralogs, and the relevant analysis, in the Results section, page 13 second paragraph.

16. Also, the reliability of crispr-cas9 system to quantify the essentiality of a gene with multiple paralogs may be biased.

This is correct in principle, but the sgRNAs that were used for the DepMap CRISPR screens have been optimized so that they do not target known paralogs. Therefore, genes with paralogs are less likely to be essential in these screens (Ito et al. Nature Genetics 2021). In fact, in order to assess digenic dependencies, defined as dependencies of targeting both paralogs simultaneously, a specialized CRISPR paralog-targeting library had to be generated (Ito et al. Nature Genetics 2021). Of note, our analyses always used the gene-level scores (rather than the guide-level scores), further diminishing the likelihood for interference between paralog genes.

17. Fix Parenthesis: '...the gene was neutral (Taylor et al. study (Taylor et al. 2018). Essentiality by CRISPR..."

This mistake has been fixed, section “Features of cancer tissues”, page 19.

**2^nd^ round**

**Reviewer 2**

The authors have very satisfactorily addressed all of my initial concerns, and I congratulate the authors again on a very interesting piece of work.

**Reviewer 3**

We thank the authors for thoroughly addressing our comments. We have one remaining comment pertaining to the data collection:
1. We note that in your response to the comments, you stated “The results originally shown in Fig. 4 were based on the combined analysis of (n=85) CRC cell lines, not to any particular cell line. Based on the Reviewer’s request below, we have recapitulated this analysis in (n=434) TCGA COAD tumors” – however, these sample size values do not appear to be in the main text or in any figures/tables. Please include a section in the methods (ideally with an accompanying table) that clarifies the following:
a. Each database that was used for data collection
b. The type of data retrieved from each database
c. The date it was retrieved
d. The number of samples that was retrieved from the database
We acknowledge that at present some of this information is scattered throughout the methods, but considering that data collection is the first step in this analysis and sample size information is quite important, it would be helpful to have this presented altogether in the first section of the methods.

**Reviewer 4**

Authors performed all the requested revisions.

1) I could not find the code repository in the manuscript. It would be helpful to the community to provide the source code to replicate the analysis results.
